# Kinetic modelling of the cellular metabolic responses underpinning *in vitro* glycolysis assays

**DOI:** 10.1002/2211-5463.13765

**Published:** 2024-01-12

**Authors:** Nitin Patil, Zohreh Mirveis, Hugh J. Byrne

**Affiliations:** ^1^ FOCAS Research Institute TU Dublin Ireland; ^2^ School of Physics, Optometric and Clinical Sciences TU Dublin Ireland

**Keywords:** extracellular acidification rate, glycolysis assay, glycolysis pathway kinetics, numerical modelling, pathway modulation

## Abstract

This study aims to demonstrate the benefits of augmenting commercially available, real‐time, *in vitro* glycolysis assays with phenomenological rate equation‐based kinetic models, describing the contributions of the underpinning metabolic pathways. To this end, a commercially available glycolysis assay, sensitive to changes in extracellular acidification (extracellular pH), was used to derive the glycolysis pathway kinetics. The pathway was numerically modelled using a series of ordinary differential rate equations, to simulate the obtained experimental results. The sensitivity of the model to the key equation parameters was also explored. The cellular glycolysis pathway kinetics were determined for three different cell‐lines, under nonmodulated and modulated conditions. Over the timescale studied, the assay demonstrated a two‐phase metabolic response, representing the differential kinetics of glycolysis pathway rate as a function of time, and this behaviour was faithfully reproduced by the model simulations. The model enabled quantitative comparison of the pathway kinetics of three cell lines, and also the modulating effect of two known drugs. Moreover, the modelling tool allows the subtle differences between different cell lines to be better elucidated and also allows augmentation of the assay sensitivity. A simplistic numerical model can faithfully reproduce the differential pathway kinetics for three different cell lines, with and without pathway‐modulating drugs, and furthermore provides insights into the cellular metabolism by elucidating the underlying mechanisms leading to the pathway end‐product. This study demonstrates that augmenting a relatively simple, real‐time, *in vitro* assay with a model of the underpinning metabolic pathway provides considerable insights into the observed differences in cellular systems.

## Graphical abstract

The kinetics of the *in vitro* glycolysis assay are deconvoluted using a simple model. The model describes the observed two‐phase metabolic response. The differential responses of three cell lines are quantitatively compared using the qualitative model. Pathway modulation by drugs is described and quantified. The potential of such models to augment the interpretation of simple assays is demonstrated.

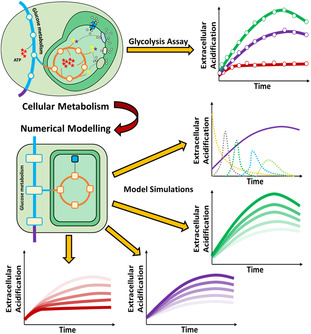

**Abbreviations**
(3,2) PG: (3,2) phosphoglycerate
1,3BPG: 1,2 bisphosphoglycerate
2DG: 2‐deoxyglucose
2DG‐P: 2‐deoxyglucose‐phosphate
Acetyl COA: acetyl coenzyme A
AMP, ADP, ATP: adenosine (mono, di, tri) phosphate
AMPK: adenosine monophosphate‐activated protein kinase
ANT: adenine nucleotide translocase
Capacity_G: glycolysis capacity
Capacity_M: mitochondrial capacity
CO_2_
: carbon dioxide
complex I: ubiquinone oxidoreductase
complex II: succinate dehydrogenase
complex III: cytochrome *c* reductase
complex IV: cytochrome *c* oxidase
CoQ: coenzyme Q; ubiquinone
cyt c: cytochrome c
DHAP: dihydroxyacetone phosphate
DMEM: Dulbecco's modified Eagle medium
ECA: extracellular acidification
ECAR: extracellular acidification rate
ETC: electron transport chain
Ex/Em: excitation/emission
F16BP: fructose 1,6 bisphosphate
F6P: fructose 6 phosphate
G6P: glucose 6 phosphate
GA3P: glyceraldehyde 3 phosphate
Glu: glucose
Glu_ex: extracellular glucose
GLUT: glucose transporters
GPI: glucose‐6‐phosphate isomerase
H: hydrogen ion
HCO_3_^−^
: bicarbonate ion
HK: hexokinase
IC: isocitrate
KG: ketoglutarate
Lac: lactate
Lac_ex: extracellular lactate
NAD: nicotinamide adenine dinucleotide
OAA: oxaloacetate
ODEs: ordinary differential equations
oligo: oligomycin
OxPP: oxidative phosphorylation pathway
PEP: phosphoenol pyruvate
PFK: phosphofructokinase
Pi: phosphate
PiC: phosphate carrier
Pyr: pyruvate
RFU: relative fluorescence units
SBML: systems biology markup language
S‐COA: succinyl coenzyme A
TCA cycle: tricarboxylic acid cycle/Kreb's cycle
TGF: time‐gated fluorescence
TRF: time‐resolved fluorescence
UV: ultraviolet

Cellular metabolism is crucial for sustaining life as we know it, and this has driven a massive interest among researchers to understand its intricacies [1]. The glycolysis pathway has attracted significant attention, in particular, as it is involved in both aerobic and anaerobic energy production, responsible for the production of precursors for other metabolic pathways, and serves as the primary energy source for cells without mitochondria [2]. Cells which have evolved to perform different specific functions throughout the body metabolise carbon at different rates, under varying circumstances, and are capable of preferentially choosing among the pathways to adapt [3, 4, 5]. The metabolic adaptations and the variance in metabolic rates have been well‐documented for disease conditions such as cancer, diabetes and neurodegenerative disorders [3], but also for healthy cells such as muscle cells, brain cells and liver cells [3]. In the context of human health and well‐being, the significance of the kinetics have been described for several disorders [6, 7, 8], and several cancer treatment strategies are already based on the metabolic kinetics [9]. In the field of metabolic engineering, understanding kinetic metabolic fluxes can guide strategies towards increased efficiency and yields, and thus have significant commercial value [10]. An understanding of the differential metabolic kinetics and the ability to interpret the cellular state based on them can thus be a powerful tool for diverse sectors of biotechnology.

Several strategies have been explored to monitor the metabolic pathway kinetics, ranging from the simple enzyme kinetic assays [11] to highly sophisticated omics approaches [12]. These strategies monitor the evolution of the components involved directly or indirectly in the metabolic process as a function of time. Fluxomics, an approach based on metabolomics, can be considered the current gold standard for monitoring metabolic pathway kinetics [13]. Metabolic flux refers to the metabolite conversion rate in a metabolic network and is a function of enzyme abundance and its activity [14, 15]. Enzyme activity is a function of the transcriptional and translational regulation along with the enzyme stability and is affected by the concentrations of enzyme, substrate, product, effector molecules, etc. [14]. While the various approaches of fluxomics specifically seek to determine the rates of metabolic reactions within a system, metabolomics provides an instantaneous representation of the metabolites in a dynamic metabolism process [14, 16]. Similar to other omics approaches, fluxomics yields a complete set of cellular metabolic fluxes but represents the phenotypic dynamics as a result of interactions of metabolome, genome, transcriptome, proteome, post‐translational modifications, environment, etc. [17]. Other omics approaches, such as genomics, transcriptomics, metagenomics, and multiomics., can be used to extend the insight into the phenotype of cellular metabolism to different levels of its regulation [14]. However, although such omics approaches have become very sophisticated and well‐adapted by the research community, a major limitation lies in the fact that they are destructive to the cell/tissue, which limits the insight provided to a snapshot of the cellular processes, and time series measurements of multiple such snapshots becomes rather laborious, costly and prone to errors [18, 19].

The simplistic kinetic glycolysis assay, marketed under different trademark names, is reported to be suitable for monitoring glycolysis rates under various cellular interventions [20]. The assay exhibits a positive luminescent response to the extracellular acidification (ECA), or the change in pH of the cellular medium, as a function of time [21].

The primary contribution to the change in extracellular pH is the increased production of lactate, the end product of the glycolysis pathway, which is secreted by the cell, along with a hydrogen ion. Further potential contributors include CO_2_, as a by‐product of mitochondrial respiration, which hydrates external to the cell to dissociate into HCO_3_^−^ and a hydrogen ion [22, 23]. The relative contribution to ECA by each of the processes varies across cell lines and is influenced by their metabolic substrates or the cellular niche [22]. Although the glycolysis assay does not discriminate the sources of acidification, it is biased in favour of sensitivity to glycolysis by incubation in the respiration buffer, and further by glucose starvation [24, 25]. The assay is marketed as suitable for obtaining important insight into the central role played by altered glycolytic activity in a wide array of physiological and pathophysiological processes [26]. The changes in luminescent signal from the assay over time can be used to calculate extracellular acidification rates (ECAR) [27]. The sensor molecule in the assay does not permeate the cell and is therefore not consumed during experimentation. Thus, it is capable of monitoring the change in ECA in real time, without interfering with the cellular metabolic processes [21]. The assay is supplied with respiration buffer, which is an unbuffered minimal medium with only one carbon source, glucose, to obtain an ECA response, which is reliant only on the supplemented glucose. The assay is relatively simplistic and can be read by commercially available fluorescence plate readers in 96‐well plates, enabling the high‐throughput measurement of multiple samples and variations of experimental conditions. To enhance the detection sensitivity, the sensor molecule is commonly designed to emit longer lived phosphorescence, which can be differentiated from any shorter lived fluorescence from the cell or medium by time gating the detection, in the so‐called time‐resolved fluorescence (TRF) mode, usually provided as an add on to fluorescence plate readers. More sophisticated instruments have been designed specifically for kinetic monitoring of cellular metabolic processes, improving sensitivity and reproducibility via optical fibre‐based optical excitation and light collection [28, 29]. The assay response as a function of time can be used to determine ECAR, and in turn be used to quantify the changes in cell metabolism as a result of drug modulators [30], varying rates in different cell lines [31], etc., and thus is a relatively inexpensive, convenient, method to monitor the kinetics of this specific metabolic process. Normally, the glycolysis assay alone is used to study the glycolysis pathway kinetics as a function of time under various well‐defined modulations, and the variations in the ECA/ECAR are correlated directly with the glycolysis pathway kinetics. Such correlation of ECA/ECAR can potentially be augmented using numerical models, which can potentially be used to predict the metabolic pathway kinetics beyond experimental sensitivity, predict the kinetics of the cascading metabolic processes leading to the end point and assign numerical values to the qualitative data for comparison.

Recent developments in computational biology have resulted in an increasing trend of integration of bioinformatics and experimental biology [32, 33, 34, 35]. The emergence of systems biology markup language (SBML) has facilitated the exchange of mathematical models of cellular processes across various platforms, which can be optimised or fitted to mimic the systemic behaviour [36, 37, 38]. Several tools such as simbiology (matlab based) [39] and celldesigner [40, 41] can aid in simplistic graphical modelling of biological pathways, which can then be underpinned by mathematical rate equations to simulate the desired evolution. The tools also provide multiple levels of control over modelling, with a wide array of simulation modalities to understand the kinetics of cellular metabolism, which might be challenging to pursue or not clearly evident in experimental studies. These modelling tools provide a convenient user interface to code the model in desired formats for easy transfer. With the rapid emergence of innovative pathway models describing systemic behaviours, several databases such as pantherDB [42] and Biomodels [38] have emerged, which can be accessed globally. Integration of cellular pathway modelling with the kinetic glycolysis assay can potentially augment the insight into the underpinning cellular metabolic pathway kinetics, to better understand differences in assay response to, for example differences in cell phenotype, or as a result of pathway modulating drugs. The models can simulate the desired cellular effect and quantify differences in rates, and therefore, for example efficacies of drugs. With increasing modelling sophistication, alongside experimental input, models can potentially be used as predictors of mechanisms of action of therapeutic agents, or toxicants.

This paper aims to demonstrate the benefits of augmenting the results of the glycolysis assay with a simplistic numerical model based entirely on rate equations, which can simulate the glycolysis pathway kinetics to reveal the underlying cascading processes from the end‐point kinetics and provide an insight into the metabolism beyond the assay sensitivity. The kinetic evolution of ECA was monitored using a commercially available kinetic glycolysis assay for three different cell lines. The choice of human lung cancer, monkey kidney and human liver cancer cell lines was purely based on the fact that they were available in house. The effect of two known glycolysis pathway modulator drugs on the evolution of ECA was then monitored as a function of time to derive the upper and lower limits of the pathway capacity. These modulators are commonly supplied with the commercial assay. Guided by the experimental observations, a simple mathematical model, based on ordinary differential rate equations, was developed to simulate the trends in ECA as a function of time for all three cell lines. The effects of the modulator drugs were incorporated into the model to reproduce the experimental results. The optimised model can faithfully simulate the differential metabolic rates, with or without modulation, and increase the insight into the cellular metabolism beyond experimental sensitivity and reproducibility.

In the next section, a brief and simplified description of the central carbon metabolism is provided, in order to clarify which aspects influence the response of the glycolysis assay used, and therefore to guide the development of a simplified, phenomenological rate equation model. For a more complete description, the reader is referred to the detailed articles dedicated to the complete cellular metabolism [43, 44] and the adenosine monophosphate activated protein kinase (AMPK) pathway crucial for maintaining the cellular energy homeostasis [45, 46].

## Cellular metabolism: a brief insight

**Fig. 1:**
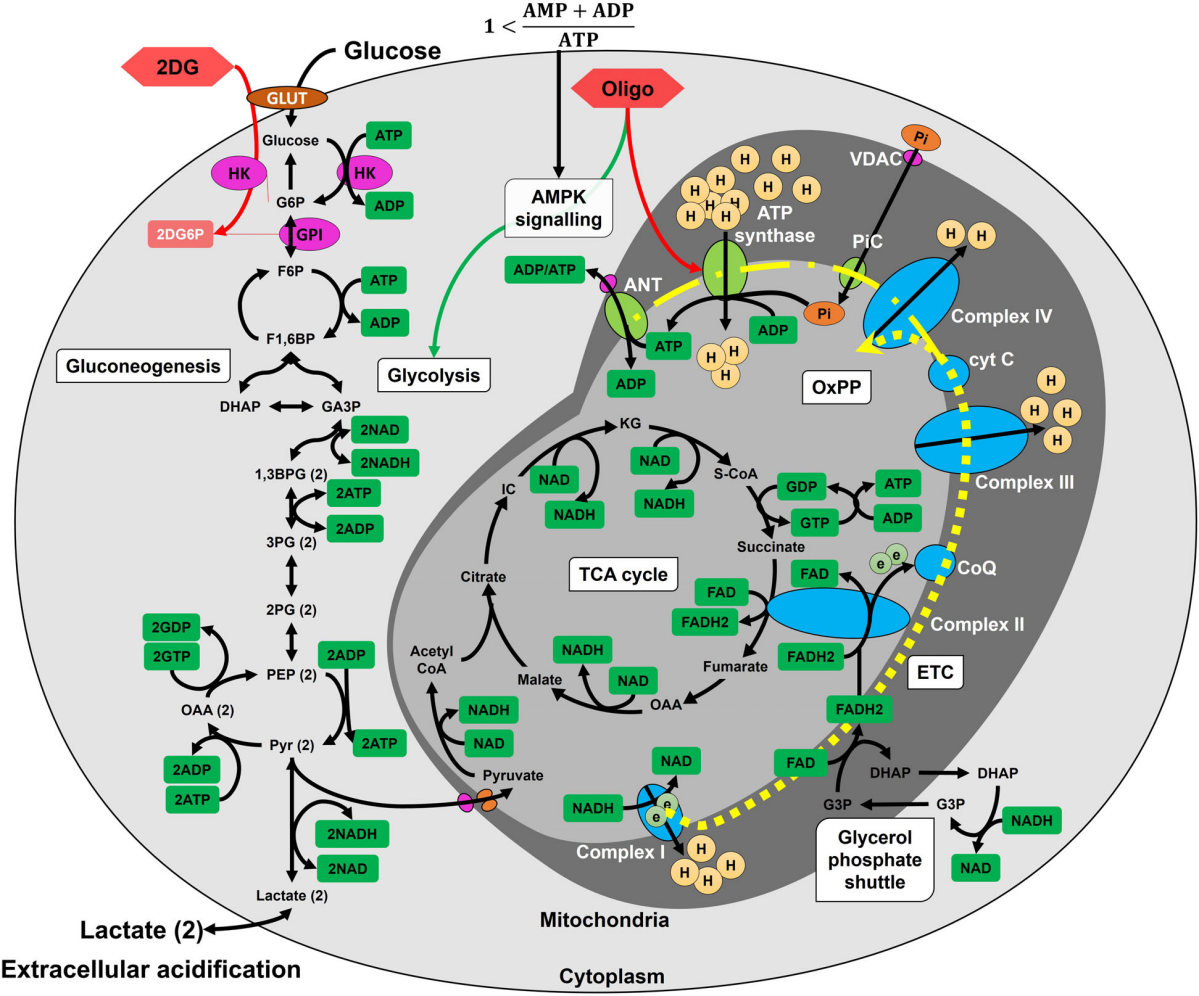
Cellular glucose metabolism: Glucose is taken up using glucose transporters (GLUT, dark orange) and first catabolised to pyruvate in glycolysis pathway (anaerobic) in the cytoplasm, which can either be reduced to lactate or enter mitochondria through voltage dependent anion channel (VDAC; pink) and mitochondrial pyruvate carriers (MPC1 and MPC2; orange) to be processed in the TCA cycle (anaerobic). Glycolysis produces two net ATP and NADH molecules. NADH can either be oxidised to NAD+ during pyruvate to lactate reduction or via the glycerol phosphate shuttle, which transfers the energy to FAD reducing it to FADH2. All the NADH and FADH2 energy molecules produced in glycolysis and TCA cycle are oxidised in the electron transport chain (ETC; blue blocks connected sequentially with yellow dashed line) which transfers protons across the inner mitochondrial membrane to the mitochondrial intermembrane space. The generated proton gradient is used to drive the ATP synthase (green block; connected to the complete OxPP with yellow line) for maximum ATP yield (29–32) per glucose monomer. Cellular energy carrier molecules are highlighted in green. 2deoxyglucose (2DG; red hexagon) is a competitive inhibitor which cannot be catabolised past 2‐deoxyglucose 6 phosphate (2DG6P; red rectangle) and accumulates in the cell, leading to the inhibition of glucose‐6‐phosphate isomerase (GPI, pink circle). Hexokinase is inhibited by the accumulation of glucose 6 phosphate (G6P), represented as a separate red line. Similarly, the inhibition of ATP synthase by oligomycin (oligo; red hexagon) is represented with a red line. Under cellular energy scarcity (equation‐top) AMPK (AMP‐activated protein kinase) pathway is activated which stimulates the glycolysis pathway. Oligomycin leads to a similar cellular energy scarcity and stimulates the glycolysis pathway (green line). Under, energy demanding conditions cells produce excessive lactate as a consequence of slow ETC response rate. When the extracellular glucose is exhausted the cells recycle lactate in TCA cycle and/or gluconeogenesis pathway. (3,2) PG, (3,2) phosphoglycerate; 1,3BPG, 1,2 bisphosphoglycerate; Acetyl COA, acetyl coenzyme A; AMP, ADP, ATP, adenosine (mono, di, tri) phosphate; ANT, adenine nucleotide translocase; complex I, ubiquinone oxidoreductase; complex II, succinate dehydrogenase; complex III, cytochrome *c* reductase; complex IV, cytochrome *c* oxidase; CoQ, coenzyme Q; ubiquinone; cyt c, cytochrome c; DHAP, dihydroxyacetone phosphate; F16BP, fructose 1,6 bisphosphate; F6P, fructose 6 phosphate; G6P, glucose 6 phosphate; GA3P, glyceraldehyde 3 phosphate; GLUT, glucose transporters; GPI, glucose‐6‐phosphate isomerase; H, hydrogen ion; HK, hexokinase; IC, isocitrate; KG, ketoglutarate; NAD, nicotinamide adenine dinucleotide; OAA, oxaloacetate; PEP, phosphoenol pyruvate; Pi, phosphate; PiC, phosphate carrier; Pyr, pyruvate; S‐COA, succinyl coenzyme A.

Cellular metabolism is a complex, highly integrated process. The integrated complexity extends beyond the metabolic pathways, to concentrations of metabolic components, signalling mechanisms, cellular growth phases, cellular physiology/pathophysiology and so on, all influencing the basic cellular metabolic processes and its kinetics [47, 48]. The most important among these influences is the cellular energetics, for which ATP (adenosine triphosphate) is the ‘energy currency’ [49]. Glucose is the primary carbon source to the cells and is involved in both aerobic and anaerobic metabolism. It is first catabolised anaerobically to pyruvate in glycolysis (Fig. 1). Normally, the pyruvate then enters mitochondria for its aerobic catabolism in the tricarboxylic acid cycle (TCA; Fig. 1). The combination of aerobic (mitochondrial) and anaerobic (glycolytic) energy production effectively yields about 29–32 ATP molecules in total per glucose molecule [50]. This process keeps the cellular [AMP + ADP]/ATP (AMP, ADP, ATP – adenosine [mono, di, tri] phosphate, respectively) ratio balanced.

However, under high energy demanding circumstances, which can occur naturally or in a diseased state, the ATP is consumed rapidly, unbalancing the ratio. This activates the AMPK (AMP activated protein kinase) signalling mechanism which positively regulates the ATP replenishing processes and negatively regulates the ATP consumption processes. Glucose being the primary carbon source to the cells the glycolysis pathway is stimulated by the AMPK signalling mechanism [45] (Fig. 1). Note that Fig. 1 only describes the AMPK signalling mechanism in general terms, as it is complex and affects multiple enzymes and transcriptional factors under various stimuli. Glucose catabolism in glycolysis yields two net ATP molecules while also reducing two NAD+ (nicotinamide adenine dinucleotide) molecules to NADH (Fig. 1). However, since the glycolytic energy production rate is much faster than the response of the electron transport chain (ETC; Fig. 1) [51, 52], the NAD+ replenishment through the ETC (Fig. 1; glycerol phosphate shuttle) cannot keep up with the pace of glycolysis, resulting in a NAD+ scarcity in the cytoplasm. Furthermore, it has been established that the activity of pyruvate dehydrogenase enzyme, which catalyses the first step of the TCA cycle (pyruvate to acetyl‐COA), remains low when the glycolytic rate is high, which leads to pyruvate accumulation in the cell [5, 53]. Both the issues of NAD+ depletion and pyruvate accumulation are resolved by converting the pyruvate to lactate while oxidising the NADH to NAD+, which can then be reused in the glycolysis pathway. The lactate produced is expelled by the cell, acidifying the extracellular medium. Once the energy ratio is balanced, the AMPK signalling mechanism is deactivated, which normalises the pathway rates. The choice of pathway under energy demanding conditions is thus a trade‐off between the high ATP yield of the mitochondrial pathway, and the rapid ATP production rate of the glycolysis pathway, of which the cells prefer the latter. Once the energy ratio is balanced, or during glucose scarcity, the cells can recycle the secreted lactate to produce more energy and cellular components in the TCA cycle or can be anabolised through the gluconeogenetic pathway to replenish cellular carbon reserves (Fig. 1).

The metabolic balance of the cell can be disrupted by several physiological stresses as well as pharmacological inducers [54]. To demonstrate the effects of such modulation strategies, this study uses the two drugs 2‐deoxyglucose (2DG) and oligomycin (oligo). The 2DG molecule is a mimic of glucose, which competes with it for phosphorylation by the hexokinase enzyme. However, the phosphorylated 2‐deoxyglucose‐phosphate (2DG‐P) cannot be utilised in the subsequent steps of glycolysis, as it inhibits glucose 6 phosphate isomerase (GPI), while hexokinase (HK) is inhibited by the accumulation of glucose 6 phosphate (product inhibition), and the combined effect leads to the inhibition of the glycolysis pathway at the very first step (Fig. 1) [55, 56, 57]. Oligomycin inhibits the ATP synthase enzyme (OxPP; Fig. 1) which shuts down the mitochondrial energy production (Fig. 1) [58]. As the mitochondrial pathway produces more ATP compared to glycolysis, its shutdown creates an energy scarcity in the cell, which activates the AMPK signalling pathway, stimulating the glycolysis pathway (Fig. 1) [58]. Oligomycin thus functions as an inhibitor of mitochondrial energy production but a stimulator to the glycolysis pathway.

## Materials and methods

### Cell culture

Three cell lines, A549 (human lung cancer), LLCMK2 (monkey kidney cells) and HepG2 (human liver cancer), were cultured in Dulbecco's Modified Eagle Medium (DMEM; 1×; Sigma Aldrich, Dublin, Ireland) supplemented with 10% MSC‐Qualified FBS (Sigma Aldrich, Dublin, Ireland) and 1% penicillin–streptomycin (Penstrep; GIBCO, ThermoFisher, Dublin, Ireland) at 37 °C in a 5% CO_2_ incubator. The A549 and HepG2 cell lines were obtained from ATCC (Manassas, VA, USA). The LLMCK2 cell line was a generous donation from U. Power, Queen's University Belfast, Northern Ireland. The cells were sub‐cultured every 3–4 days, when their confluency reached 60–80%. For subculturing, briefly, the growth medium was removed, and cells were washed with 10 mL of Dulbecco's Phosphate‐Buffered Saline (D‐PBS, containing no calcium, magnesium, or phenol red; Sigma Aldrich) to remove any remnant medium and cell secretions. Then, cells were trypsinised (Trypsin; GIBCO, ThermoFisher), seeded at the appropriate volume (1–1.5%) and further incubated at 37 °C in a 5% CO_2_.

### Assay

The pH‐Xtra glycolysis assay was purchased from Agilent, Dublin, Ireland. The recipe for respiration buffer was reproduced from the respiration buffer provided with the assay and prepared in house, without glucose: 1 mm K‐phosphate, 70 mm NaCl, 50 mm KCl, 0.8 mm MgSO_4_, 2.4 mm CaCl_2_. The luminescent sensor molecule, supplied as the pH‐Xtra reagent, allows direct, real‐time, kinetic measurement of ECA or the change in pH as it is cell impermeable and it is not consumed during experimentation [21]. It exhibits a positive signal response across the biological range for the analysis of ECA [21]. Rates of extracellular acidification (ECAR) can be calculated from the changes in assay response over time [21].

At approximately 80% confluency, cells were trypsinised and harvested. The cells were seeded at 6.5 × 10^4^ cell density in a flat bottomed, white, 96‐well plates with 200 μL DMEM and incubated at 37 °C in a 5% CO_2_. After 14–16 h, when the cells were attached to the bottom of the plate, they were washed twice with glucose‐free respiration buffer and incubated in 100 μL glucose‐free respiration buffer at 37 °C in a CO_2_ free incubator for 2 h. Incubation in CO_2_ free incubator was as described by the assay supplier. To reduce ECA by cellular respiration, the process was modified further by incubating the cells in glucose free respiration buffer to effectively starve the cells and cause them to consume their carbon reserves. This essentially reduces the acidification signal by cellular carbon reserves as observed in Fig. S1. After incubation, the glucose‐free respiration buffer was replaced with 90 μL respiration buffer containing 20 mm glucose and 10 μL pH‐Xtra reagent was added.

The plate was immediately placed in the GENios microplate reader (Tecan, Grodig, Austria) without lid (to avoid accumulation of condensation moisture), set to kinetic monitoring in single‐read Time Resolved Fluorescence (TRF) mode at Ex/Em 360/595 nm, temperature 37 °C, top read mode with 100 μs lag and 100 μs integration time. The instrument was set for 400 cycles for 140 min (0.35 min per cycle). The gain value was automatically set to 176 for the first experiment and was maintained for subsequent experiments.

For pathway stimulation and inhibition experiments, 2 μm and 100 mm oligomycin and 2DG, respectively, were added to the mix while keeping the overall volume in the well constant.

The assay response, as a measure of the ECA, was monitored under conditions of control, inhibition by 2‐deoxyglucose (2DG) and stimulation by oligomycin (oligo) for up to 140 min for the three different cell lines, each in triplicates. These data were normalised by the cell‐free signal control to eliminate any measurement drift over time. The average values from replicates were used for data fitting in the model. All the experiments were performed in parallel, on the same 96‐well plates, for ease of comparison. Note, although the TRF mode measures delayed fluorescence or phosphorescence, literature from the assay supplier and publications based on the assay commonly plot the assay response as Fluorescence, in relative fluorescence units (RFU). This convention is adopted here.

### Modelling

Since the glycolysis assay provides only the kinetic evolution of the ECA, a model based on simple (ordinary differential) rate equations was established, to better elucidate the underpinning metabolic processes. Two modelling tools were explored, namely celldesigner [40, 59] and simbiology [39]. In terms of modelling, both tools are very similar and provide similar functionalities. celldesigner is an open source platform for modelling, resulting in codes of .xml format and provides the functionality to export the model to different levels and versions of .sbml format. An advantage of using this tool is that it can interact with several databases for importing specific details or even complete models. This tool was used to evolve our understanding of modelling from the databases. simbiology, on the other hand, is a matlab‐based application, which can actively interact with matlab, thus adding to its functionality. simbiology codes the model in .sbproj, a matlab format, but can export it in .sbml format. As this tool inherits matlab functionalities, it can save and run multiple programs in parallel, import experimental data, overlay the data and even fit the data by the model. The simplistic mathematical model for the glycolysis pathway was developed in simbiology ver.6.3 (Fig. 3). Modelling was primarily performed on a Dell OptiPlex 5060 and Lenovo ideapad Flex 5‐14ALC05‐Type 82HU, and in all cases was complete in a matter of seconds. Model structure, equations and parameters are discussed in detail in the Results section.

## Results

### Kinetic assay to monitor extracellular acidification as a function of time in cell lines

The nonpermeating pH Xtra glycolysis assay reagent monitors ECA as a change in pH of the cell culture medium as a result of cellular lactate production, in the form of a (fluorescence/phosphorescence) light signal whose increase/decrease can be monitored as a function of time to provide insight into the glycolytic rate. The assay signal was monitored in real time for up to 140 min for the three different cell lines, A549, LLCMK2 and HepG2.

**Fig. 2:**
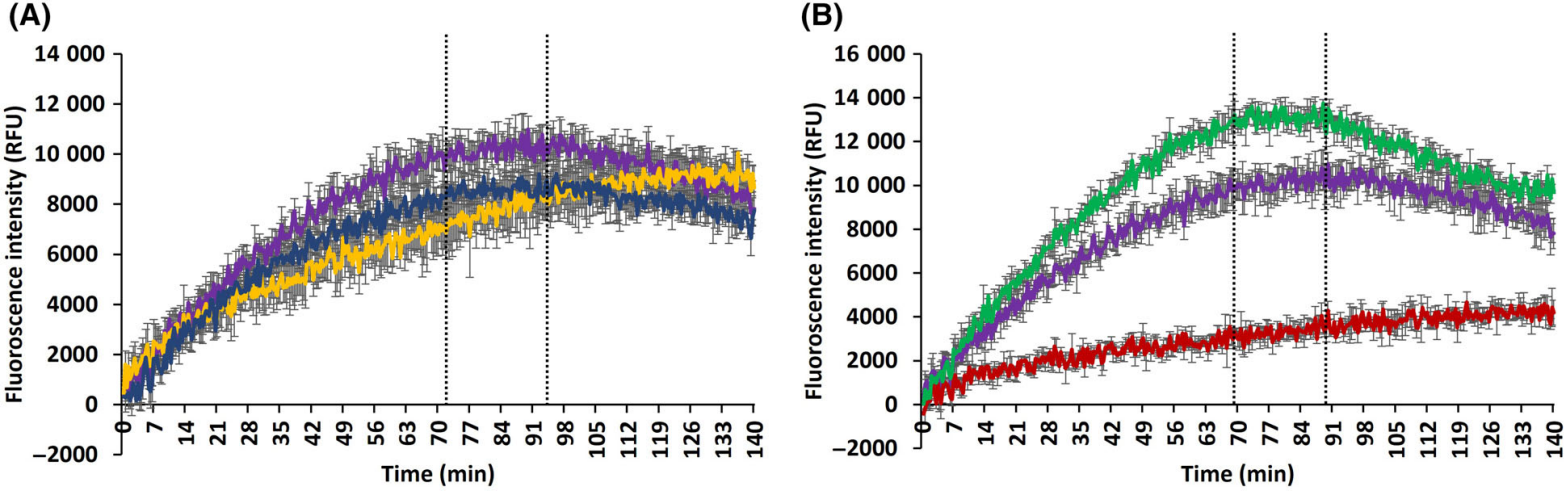
(A) Assay response as a function of time (up to 140 min) representative of extracellular acidification (ECA) for A549 (purple), LLCMK2 (blue), and HepG2 (yellow) cell lines; and (B) ECA for A549 control (purple), stimulated by oligomycin (green), and inhibited by 2‐deoxyglucose (red). The dotted vertical lines approximately delineate the two response phases, < 70 min, and > 90 min. Note: the control assay responses (purple) for A549 cell lines in ‘A’ and ‘B’ are the same.

As observed in Fig. 2A (purple), ECA for the A549 cell line initially increases rapidly up to ~75 min, at which point it starts to saturate. After ~95 min, a decrease in the ECA is observed. As the pH Xtra reagent is not consumed during the measurement, this decrease in ECA levels is indicative of reduction of extracellular pH and/or consumption of lactate by the cells as, described in Materials and methods section. A similar trend was observed in the LLCMK2 cell line (Fig. 2A, blue), whereby the ECA increases rapidly up to ~75 min, before saturating and decreasing after ~95 min. The observed levels of ECA were lower for the LLCMK2 cell line compared with the A549, however, indicative of lower ECAR. For the HepG2 cell line (Fig. 2A, yellow), an increase of ECA was observed, which only begins to saturate at about ~125 min. Any further decrease, as observed for the other two cell lines, was beyond the experimental time bounds.

Furthermore, the cellular glycolysis pathway rates were modulated using two drugs with well‐known modes of action (Fig. 1). 2DG, a competitive inhibitor of the hexokinase and glucose 6 phosphate isomerase enzyme in the glycolysis pathway, effectively reduced the ECAR in the A549 cell line, as shown in Fig. 2B (red). On the other hand, Oligomycin, which acts on ATP synthase enzyme and blocks mitochondrial energy production, can be seen to stimulate the glycolysis pathway, as shown in Fig. 2B (green). In the stimulated condition, a similar rapid increase in ECA up to ~70 min can be observed, which then saturates and begins to decrease rapidly at ~90 min, similar to the control condition (Fig. 2B, green), although the overall assay response has increased, because of increased response rates. Inhibiting the glycolysis pathway, the overall response rates have been reduced, and, as a result, the ECA is seen to steadily increase, only just reaching a plateau within the timescale of the measurement. The gradual increase in the ECA response upon inhibition is consistent with other reported trends of post‐2DG acidification, attributed to nonglycolytic/mitochondrial or residual glycolysis after inhibition. Inhibition is usually observed as a steep decline in the proton efflux rate which stabilises, but at a nonzero value [60, 61].

Applying similar modulation to the ECAR analysis of the LLCMK2 and HepG2 cell lines, a similar trend as that of the A549 cells was observed for the LLCMK2 cell line (Fig. S2A). For the HepG2 cell line, a similar trend was observed for the inhibition, although, oligomycin did not produce a pronounced stimulating effect, as observed by the overlapped ECA curves for control and simulation (Fig. S2B).

The observed trends in ECAR are similar to those reported by Niklas *et al*. [5], which also indicate two metabolic phases; the first is one of high glycolytic activity, high lactate production and low to no TCA/mitochondrial activity, while the second phase is characterised by low glycolytic activity, no lactate production, high lactate consumption, and increased TCA activity. It is important to note, however, that, since the study by Niklas *et al*. was conducted over a longer timeframe (160 h), and the cells were incubated in very different media, the behaviour is not directly comparable to that observed in the respiration buffer of the commercial glycolysis assay. Nevertheless, both are indicative of the underlying metabolic adaptations in the cellular cytoplasmic and mitochondrial metabolic processes, as glucose becomes exhausted and lactate is consumed.

Furthermore, in a study which used defined buffer medium (DMEM Sigma Aldrich D5030‐HEPPS) within a comparable time frame to that of the glycolysis assay, it has been shown that oligomycin induces proton uncoupling in the mitochondrial membrane in the presence of pyruvate and lactate, which restores mitochondrial oxygen consumption, thereby indicating active proton transfer across mitochondrial inner membrane even though ATP synthase enzyme is inactive [62]. Additionally, it was shown that lactate stimulates its own consumption as a preferential bioenergetic substrate in the mitochondria, as long as sufficient oxygen is available, and some ETC functionality (proton transfer across inner membrane) is preserved [63]. This was validated using complex I, complex III inhibitors along with cell lines with partially and completely inactive ETC functionality [63], and validates the observed lactate uptake by the cells in the presence of the glycolysis pathway stimulator drug oligomycin. Furthermore, the study by Hearne *et al*. [62], also indicates that the proton uncoupling is reversed in the presence of glucose in the medium, as the cells favour the glycolysis pathway to restore the ATP deficit. This support the notion that the glucose is exhausted at the peaks of the ECA response curves in control and oligomycin stimulated experiments, resulting in lactate consumption, as evident in the subsequent decrease in ECA response. Additionally, it should be noted that, once consumed by the cells, if all the lactate was catabolised in the TCA cycle, the resultant generation of CO_2_ would result in further ECA, and thus, the assay should not show a reduced response. Alternative and/or additional fates of lactate within the cell may therefore come into play [64], although, for the sake of simplicity of the model, all the flux is assumed to enter the TCA, as the CO_2_ acidification is not accounted in the model (Next section: Mathematical modelling of cellular glucose metabolic pathway).

The ECA assay thus ready provides real‐time, *in situ* measurement of the lactate production by the cell population, which is the result of the glucose metabolism. The ECA profiles of the A549 and LLCMK2 cells are consistent with two phases of the cell metabolism, while the differences between the cell lines indicate different ECAR, which are in turn indicative of different rates of the underlying metabolic processes in the different cells. Modulation of these rates is achieved in part by the addition of pathway inhibition or enhancement, which is seen to result in a well‐defined change in the assay output, albeit the impact is different in the different cell lines. Note that the lack of observable enhancement for the case of the HepG2 cell line was not expected and may be the result of mutations of the in‐house cell line over the course of several cell line passages. Nevertheless, these data serve as valuable illustration of varying cell lines responses, due to different metabolic response rates. Critically, the differences in the ECA profiles for the different cell lines, and under the conditions of inhibition and enhancement, are a manifestation of differences in the kinetics of the underpinning metabolic response processes, which, although complex, can be modelled to considerably enhance the understanding of the processes and the implications of the observed differences.

### Mathematical modelling of cellular glucose metabolic pathway

**Fig. 3:**
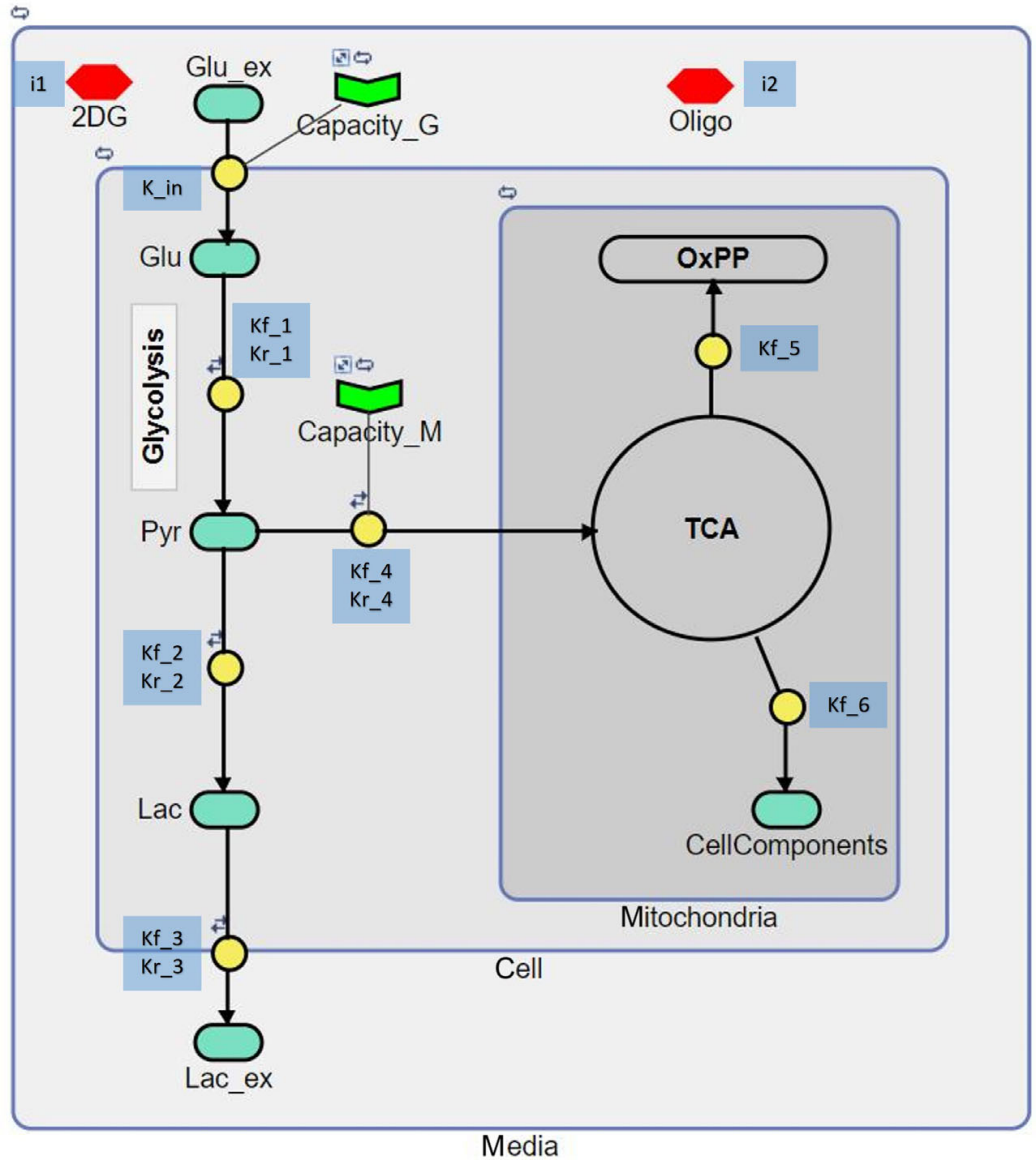
Metabolic pathway model: Model components (light green) are connected according to the reactions stated in Table 1 through reaction blocks (yellow). The components were compartmentalised (grey compartments) according to the pathway location in cells. Reaction rates (k_in (input), kf (forward), and kr (reverse)) involved in different reactions are written beside their respective reaction blocks (blue boxes). Drugs are represented by red hexagons. Pathway capacities are indicated by green chevrons. The TCA and OxPP are considered as model components but represented differently as hollow circles. Every arrow represents a reaction rate and the icons ‘
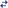
’, ‘
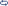
’ and ‘
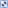
’ on the reaction blocks or components represent reversibility, constant and steady state respectively.

The process of cellular glycolysis under the conditions of the commercial assay, leading to lactate production and ECA, was modelled using the simbiology tool. The model is based entirely on ordinary differential rate equations, which are described in Table 1, and is shown schematically in Fig. 3. Each line connecting two or more components through a reaction block (yellow circles) in the model represents transition of metabolites with an assigned rate equation. Overall, the model comprises of seven equations of which four reactions are reversible, as described by ‘
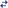
’ symbol over reaction blocks. The model components are compartmentalised according to their metabolic pathways and cellular localisation. The glycolysis pathway, represented by glucose (Glu), pyruvate (Pyr) and lactate (Lac) are localised in the ‘cell’ compartment, that is, the cytoplasm of the cell. Since, the assay is limited to the insight into the glycolysis pathway, the mitochondrial compartment was minimalised by considering the complete tricarboxylic acid cycle (TCA) and the oxidative phosphorylation pathway as a single metabolite (OxPP). Cellcomponents block localised in the mitochondria are a representation of the metabolic pathway by‐products, such as amino acids and nucleic acids. The biggest compartment, ‘medium’, is comprised of a Glucose reservoir (Glu_ex), which feeds into the ‘cell’ compartment with the rate ‘k_in’, regulated by the glycolysis capacity (Capacity_G), as described by Reaction 1. The glycolytic capacity aids in regulating the influx of glucose into the cell and is an overall representation of cellular glucose transport which allows regulated influx of glucose to the cell and prevents uncontrolled flooding of the cell with glucose [65, 66, 67, 68]. This regulation was incorporated into the rate equations as ‘Capacity_G – Glu’, which increases the glucose inflow at low intracellular concentrations, but reduces it as the internal concentration increases, and essentially nullifies the rate when the intracellular glucose (Glu) value reaches the Capacity_G. The internalised glucose ‘Glu’ is further reversibly catabolised to pyruvate, ‘Pyr’, with rates kf_1 (forward) and kr_1 (reverse), as described by Reaction 2. ‘Pyr’ then can be reversibly catabolised to lactate, ‘Lac’, with rates kf_2 (forward) and kr_2 (reverse) (Reaction 3), or reversibly enter the ‘TCA’ cycle localised in the ‘mitochondria’ with rates kf_4 (forward) and kr_4 (reverse) (Reaction 5). The influx of ‘Pyr’ into the mitochondria is regulated by the mitochondrial capacity (Capacity_M) which, similar to ‘Capacity_G’, is introduced to regulate the forward flux (i.e., the ‘Capacity_M’ only regulates the flux entering mitochondria not the other way around). The mitochondrial capacity has two primary roles, firstly, when the cells are catabolising glucose it keeps the feed towards the TCA low (also observed in previous studies [4, 5]) and secondly it aids in rapid uptake of lactate into the TCA. The generated ‘Lac’ can then be reversibly transported to Lac_ex in the media compartment outside the cell, with rates kf_3 (forward) and kr_3 (reverse), as described by Reaction 4. The flux in the ‘TCA’ ultimately ends up either reverting to glycolysis or unidirectionally into the ‘OxPP’ or ‘CellComponents’ with rates kf_5 (forward; Reaction 6) and kf_6 (forward; Reaction 7) respectively.

**Table 1:** (Top) Reactions used in the model, including their reversibilities and their respective rate equations. (Bottom) The generated resultant ordinary differential equations from the equations for the model.

Sr. no.	Reaction	Rate equation
1	Glu_ex + Capacity_G → Glu	k_in*Glu_ex*(Capacity_G‐Glu)
2	Glu ↔ Pyr	kf_1*Glu*(1 + Oligomycin*i2‐[2DG]*i1)‐kr_1*Pyr
3	Pyr ↔ Lac	kf_2*Pyr − kr_2*Lac
4	Lac ↔ Lac_ex	kf_3*Lac − kr_3*Lac_ex
5	Pyr + Capacity_M ↔ TCA	kf_4*Pyr*(Capacity_M‐TCA)‐kr_4*TCA
6	TCA → OxPP	kf_5*TCA*(1‐Oligomycin*i2)
7	TCA → CellComponents	kf_6*TCA

Sr. no.	ODE's
1	d(Glu_ex)/dt = 1/media*(−(k_in*Glu_ex*(Capacity_G‐Glu)))
2	d(Lac_ex)/dt = 1/media*((kf_3*Lac‐kr_3*Lac_ex))
3	d(Glu)/dt = 1/cell*((k_in*Glu_ex*(Capacity_G‐Glu)) − (kf_1*Glu*(1 + Oligomycin*i2‐[2DG]*i1)‐kr_1*Pyr))
4	d(Pyr)/dt = 1/cell*((kf_1*Glu*(1 + Oligomycin*i2‐[2DG]*i1)‐kr_1*Pyr) − (kf_2*Pyr‐kr_2*Lac) − (kf_4*Pyr*(Capacity_M‐TCA)‐kr_4*TCA))
5	d(Lac)/dt = 1/cell*((kf_2*Pyr‐kr_2*Lac) − (kf_3*Lac‐kr_3*Lac_ex))
6	d(TCA)/dt = 1/mitochondria*((kf_4*Pyr*(Capacity_M‐TCA)‐kr_4*TCA) − (kf_5*TCA*(1‐Oligomycin*i2)) − (kf_6*TCA))
7	d(OxPP)/dt = 1/mitochondria*((kf_5*TCA*(1‐Oligomycin*i2)))
8	d(CellComponents)/dt = 1/mitochondria*((kf_6*TCA))

The compartment values, and Capacity_G and Capacity_M values were kept constant, that is they do not change even when they were incorporated in the reaction and represented by ‘
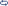
’ symbol over components/compartments. Finally, the Capacity_G and Capacity_M were also set as boundary conditions, as they are incorporated for control of flux rather than a consumable component in the model represented by ‘
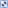
’ over the respective blocks. Each of the model compartment values, representing their population or capacity is set to a constant by default, but can be set to any value and can also be variable. This essentially allows to compensate for the number of the cellular compartments, and it is incorporated into the ordinary differential equations (ODEs) as the inverse of the compartment value (Table 1). This functionality was not utilised in the presented model.

2‐Deoxyglucose (2DG) and oligomycin (Oligo) drugs used to modulate the pathway flux in experiments were also incorporated into the model. 2‐Deoxyglucose is a competitive inhibitor of hexokinase and glucose‐6‐phosphate isomerase enzymes, which blocks the glycolytic flux at the very first step in the pathway. 2DG was thus included in Reaction 2 as an inhibitor acting with a rate of ‘i1’, the conversion of glucose to pyruvate (Materials and methods section, Fig. 1). The inhibition was formulated as ‘1‐inhibitor concentration*inhibition rate’ which multiplies into the forward rate of Reaction 2. This essentially subtracts the inhibition rate from the forward flux in the reaction. Oligomycin has the effect of stimulating the glycolysis pathway, by inhibiting the ATP synthase enzyme in the OxPP, blocking the mitochondrial ATP synthesis (Materials and methods section, Fig. 1). This imbalances cellular the ADP/ATP ratio, stimulating the glycolysis pathway. This inhibition by oligomycin is included in Reaction 6, which blocks the flux towards the OxPP at a rate ‘i2’. The unbalanced cellular ADP/ATP ratio further activates the AMPK signalling pathway, which in turn inhibits cellular processes such as growth, fatty acid oxidation and gluconeogenesis, but also stimulates the PFK enzyme directly, and several glycolysis facilitators indirectly through transcriptional factors, such as glucose transport, hexokinase and lactate dehydrogenase [69, 70, 71]. The AMPK pathway tackles the induced energy crisis until the ADP/ATP balance is stabilised, but there are indications of other persisting mechanism, which maintain the high glycolytic rate for an extended period after the balance is restored [58]. Hence, in the model, oligomycin was also incorporated as a stimulator at Reaction 2, which is merely a minimalistic representation of the niche effect of the AMPK signalling pathway phenotype (Materials and methods section, Fig. 1). Although, it should be noted that the inhibition at Reaction 6 is sufficient to mimic the increased lactate production, the stimulation by oligomycin added at the glucose‐pyruvate node (Reaction 2) is to keep the variance in the rates of intermediate steps true to reality (i.e., it increases glucose uptake rate thereby mimicking the true intracellular behaviour). To reproduce the effect of stimulation, unlike inhibition, the rate adds into the reaction rate as ‘1+ stimulator concentration *stimulation rate’. Although, the drugs were incorporated into the rate equations of their respective sites of activities, they could not be connected with reaction lines in Fig. 3, as the tool incorporates all the components connected through reaction blocks into a reaction, which influences its kinetic evolution even if the component value is set to zero. The reactions with their rate equations and the overall ODEs for the model are as described in Table 1.

### Simulating experimental ECA assay response curves

**Fig. 4:**
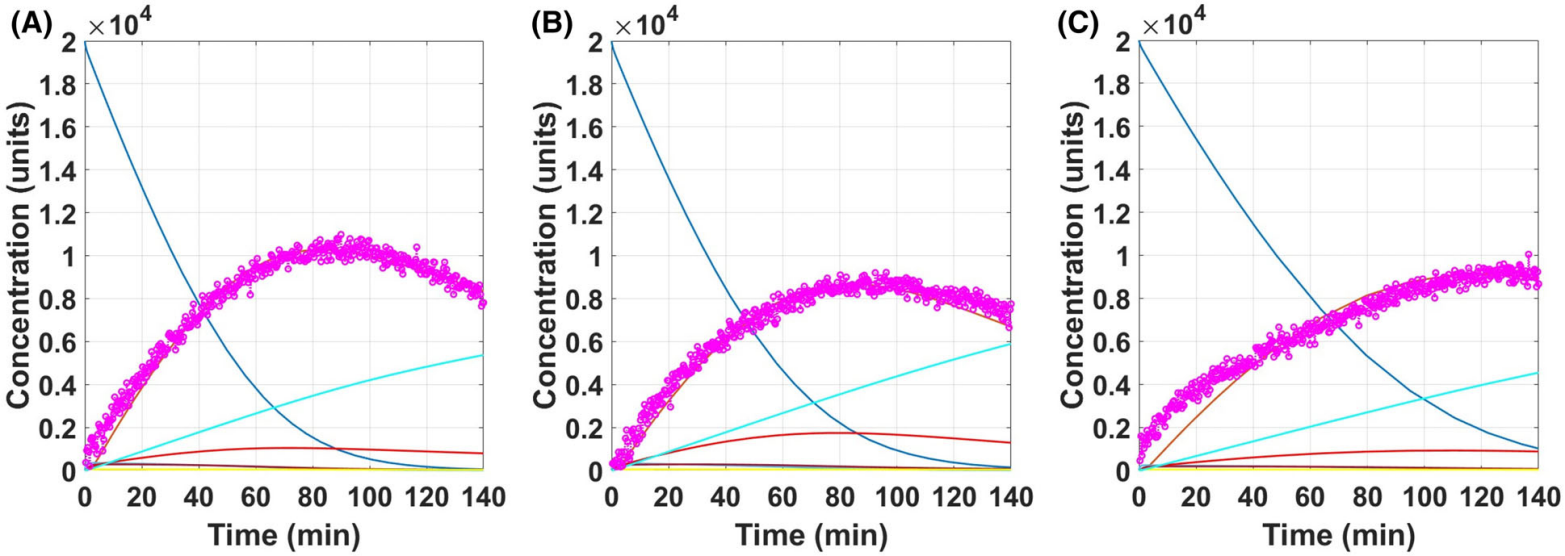
Model simulation with the optimised parameters for A659 cell line (A), LLCMK2 cell line (B), and HepG2 cell line (C). The respective experimental ECA curves (pink) are overlaid on the model to represent the optimal simulation. The Glu_ex reservoir (dark blue) is steadily consumed and the Lac_ex (orange) is seen to increase concomitantly. The CellComponents (light blue) and OxPP (green) lines overlap and increased gradually. As the glycolytic rate was very high, the evolution of intermediate metabolites (Glu—moderate blue; Pyr—maroon; Lac—red; TCA—yellow) was very rapid and thus not discernible at this scale.

Initially, the ECA data obtained from the A549 cell line were used for data modelling, as it had the highest response rate among all three cell lines. This provided a basis for initial optimisation of values of rate constants and components, which could then be reduced for simulating the ECAR profiles of the other cell lines. The experimental ECAR curve for A549 sample overlaid on the optimised simulated data is shown in Fig. 4. The optimised parameters obtained by data fitting are as described in Table 2.

**Table 2:** Optimised values for model species/components (left) and the rate constants (right) for A549 cell line. The species are segregated according to their parent compartments and the rate constants belonging to the same reaction are colour coded.

Species			Rate constants		
Sr. no.	Name	Value	Sr. no.	Name	Value
**Media**			1	k_in	0.00012
1	Glu_ex	20 000	2	kf_1	1
2	Lac_ex	0	3	kr_1	0.01
3	Capacity_G	500	4	kf_2	1
4	Oligomycin	0	5	kr_2	0.1
5	2DG	0	6	kf_3	1
**Cell**			7	kr_3	0.1
1	Glu	0	8	kf_4	0.1
2	Pyr	0	9	kr_4	1
3	Lac	0	10	kf_5	1
4	Capacity_M	50	11	kf_6	1
**Mitochondria**			12	i1	0.1
1	TCA	0	13	i2	0.1
2	OxPP	0			
3	CellComponents	0			

As only glucose was fed to the cells during the assay measurement, the quantities of all cellular components were set to zero, under initial conditions, with the exception of Glu_ex, which was set to a concentration of 20 000, as a representation of the 20 000 μm glucose fed to the cells to initiate the assay response. Rate constants thus have units of molar mass per unit time (μm·min^−1^). Simulating the first phase of increasing ECA observed in the experimental curve (Fig. 2) was mainly dependent on the value of ‘Capacity_G’, as it regulated the amount of glucose entering the cell from the reservoir. Further down the pathway, high forward rates (kf) and low reverse rates (kr) of the intermediate nodes of the glycolysis pathway ensure no accumulation of the flux at the intermediate steps. Additionally, the flux towards mitochondrial ‘TCA’ is limited by the Capacity_M and the high reverse and low forward rates of Reaction 5. These factors influenced the simulation of the experimental phase one and ensured a rapid glucose to lactate turnover. Since the study focusses on the extent of insight obtained using the glycolysis assay, which is limited to the end‐point kinetics and the only known value is the initial glucose concentration, all the values described in Table 2 for rate constants including the capacities G and M were based on the best fit of the ECAR. Experimentally, the observed rate will also depend on the cellular density, even for the same cell line, and on cellular phenotype, among other factors, and therefore, it is difficult to compare values here to literature values. The model applied to the assay results across a range of different cell types, and inhibition/promotion conditions should be mutually comparable, however.

Similarly, to simulate the second phase of the experimental curve, in which the ECA levels are seen to decrease (Fig. 2), it was seen that the reversible transport of lactate by the cells was critical. As all the glycolytic intermediates had high forward rates and low reverse rates, the lactate cannot be redirected towards glucose (gluconeogenesis). Although slower, the TCA cycle can uptake this carbon flux, as it has two end‐products (OxPP and CellComponents), which are not reversible. When the glucose reservoir is almost empty, the glycolytic forward rate drops, which promotes the lactate reverse rate, feeding Lac_ex back in to the cell, towards the mitochondria, but regulated by Capacity_M. Hence, the Capacity_M mainly influences the simulation of the second phase observed in the experimental curve.

In fact, lactate can also enter the gluconeogenetic pathway for glucose anabolism [64], but as this is a closed cycle, it is ultimately consumed via the TCA cycle because of its complexity. The gluconeogenetic path taken by lactate was neglected in the present model, which rather focused only on the TCA cycle to mimic the two phases with only one model, while maintaining constant rates throughout the simulation time.

Thus, the model reproduces the two phases of initial increase, followed by reduction in ECAR, observed experimentally and described by Niklas *et al*. [5]. The first is characterised by high glycolytic activity and lactate production while the TCA activity is low. Then, the model enters the second phase as the glucose reservoir is exhausted, resulting in reduced lactate production but high lactate consumption via the TCA cycle.

Once the model was optimised to simulate the ECAR response of A549 cell line, the parameters for the other two cell lines were optimised, with the minimal number of changes in parameters, rate, to observe what elements were critical to represent a certain metabolic phenotype.

The LLCMK2 cell line has a very similar ECAR curve to that of the A549 cell line, but the rate of glucose consumption is lower. This effect can simply be mimicked by increasing the rate of reversibility ‘kr_3’ for Lac to Lac_ex node to 0.2 from 0.1 and reducing the k_in from 0.00012 to 0.0001. Similarly, for the HepG2 cell line, the glucose consumption rate is much slower, and the lactate consumption step is significantly delayed. This effect was mimicked by reducing the glycolytic capacity by 150 units to 350 to reduce the glycolytic rate. The mitochondrial capacity was also reduced by 10 units to 40 to slow down the mitochondrial glucose uptake, and k_in was reduced by 0.00002 to 0.0001.

The overall modifications made to the model to simulate the ECAR responses of the two cell lines are summarised in Table 3.

**Table 3:** Model optimisation for LLMCK2 and HepG2 cell lines: The columns with ‘same’ written in them indicate the values are same as optimised for A549 cell line (Table 2).

Model parameters				
Sr. no.	Name	Value		
		A549	LLC‐MK2	HepG2
1	k_in	0.00012	0.0001	0.0001
2	Capacity_G	500	500	350
3	Capacity_M	50	50	40
4	kr_3	0.1	0.2	0.1
5	i2	0.1	0.1	0.01
All other parameters are the same as A549				

### Modelling the glycolysis pathway modulations

One of the best adapted use of the kinetic glycolysis assay is to study the change in rate of the metabolic process as a result of different modulations [30, 72]. The response of the assay to two well‐known modulators of the glycolysis pathway (2DG‐inhibitor and oligomycin‐stimulator) were investigated to test the ability of the model to reproduce the effect observed experimentally (Fig. 2B).

**Fig. 5:**
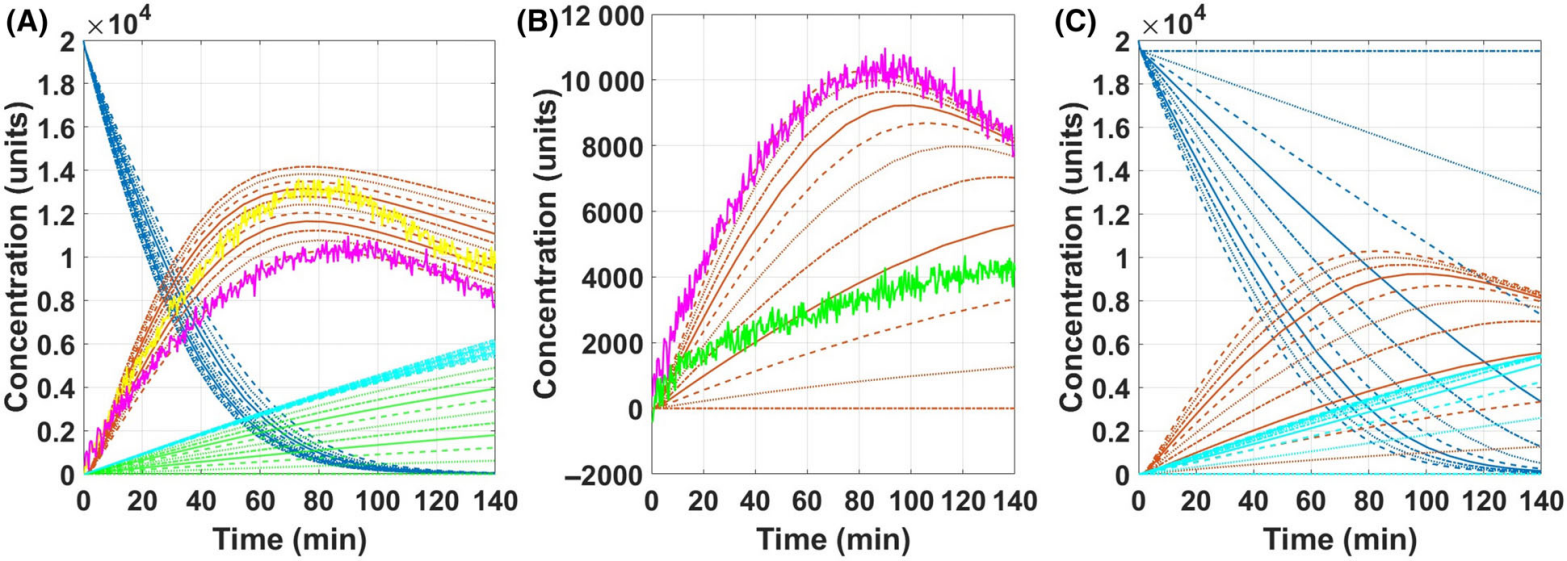
Pathway modulation using (A) oligomycin and (B) 2DG for the A549 cell line optimised model: The experimental extracellular acidification (ECA) results, pink, yellow and green lines representing control, stimulated and inhibited conditions, respectively (from Fig. 2B), are overlaid on top of the simulated lactate (orange) curves. The evolution of extracellular glucose (dark blue) and the model end‐products OxPP (green) and CellComponents (light blue) are also shown. The extracellular glucose and model end‐products are shown separately for inhibition by 2DG in ‘C’ for better visualisation of extracellular lactate curves. Note: the control experimental responses (pink) in ‘A’ and ‘B’ are the same.

Using the inbuilt functionality to systematically vary the starting parameters in the simbiology tool, the response with different concentrations of modulators, ranging for 0–10 units, was simulated (Fig. 5). As observed in Fig. 5A, by increasing the oligomycin glycolysis pathway stimulator gradually, increasing lactate production rates are observed. This effect is also reproduced in the glucose consumption rate, as it gradually increases with increasing oligomycin concentration. Furthermore, with increasing concentration, a reduction in the OxPP rate is observed, while there is a subtle increase in the CellComponents. When a similar simulation was performed with 2DG, a drastic drop in the extracellular lactate production with increasing drug concentration was observed (Fig. 5B,C). A similar drop is observed in glucose consumption rate and the OxPP and CellComponent formation (Fig. 5B,C). As the modulator has no effect on the OxPP or CellComponent formation rates, the graphs overlay for their evolution.

The model faithfully reproduces the inhibitory and stimulatory effects of the two drugs on the assay response for the A549 cell line. Similar simulations were performed for the LLCMK2 cell line and the modulators showed a similar response to that of A549 cell line, with an increased inhibition rate i2 (Fig. S3). As the HepG2 cell line did not show any effect of oligomycin (stimulator), the rate of inhibition (i2) was reduced to 0.01. This reproduced the effect of no stimulation by oligomycin but inhibition by 2DG (Fig. S4).

The optimised parameters for all three cell lines necessary to simulate the control along with the modulations are compared in Table 3.

### Model sensitivity analysis

Once the model was constructed and optimised to reproduce the experimental assay response data, a sensitivity analysis was performed to identify the effect which variations on the primary elements of the pathway have on the overall model output. For the sensitivity analysis, the effect of 10% in increment or decrement from the optimised values of each parameter of the model for the A549 cell line on the model performance was evaluated independently, for 11 consecutive simulations (five increase and five decrease). The results of the sensitivity analysis are provided in Figs S5–S11.

Varying the value of ‘Glu_ex’ (extracellular glucose) resulted in increased lactate production with each increment. The rate of lactate production in the first phase or consumption in the second remain constant. The glucose consumption rate is similar in all variations but towards the end of glucose consumption (in the second phase) the glucose uptake rate reduces with reducing concentration, as the system shifts to lactate consumption. The glucose is exhausted at the same timepoint in all variations (Fig. S5A).

Changes in the glucose uptake rate constant ‘k_in’ have a minor effect on the lactate production and glucose consumption rates, both increasing. The point at which glucose and the lactate are exhausted is unchanged, indicating the rate constant can only modulate the first phase (Fig. S5B).

‘kf_1’ has a similar but slightly more pronounced effect than ‘k_in’ (Fig. S6A). ‘kr_1’ on the other hand has no effect on the lactate production or glucose consumption, as the initial value is significantly lower than the corresponding feed forward rate (Fig. S6B).

‘kf_2’, ‘kf_3’, ‘kr_2’, ‘kr_3’ similarly have no effect on glucose consumption rates. With increasing ‘kf_2’ and ‘kf_3’ values, slightly increased lactate production rates can be observed, as evident from the slight increments in the lactate peaks. As these reactions are involved in the balance between the two side of metabolism, an inverse effect on lactate consumption (i.e. reduced lactate consumption with increased values) can be observed, as evident from the spreading of the curves in the phase two (Figs S7A and S8A). Similarly, as ‘kr_2’ and ‘kr_3’ are the reversibility rates of ‘kf_2’ and ‘kf_3’ respectively, they show an exact opposite effect to the feed forward counterparts (Figs S7B and S8B). The effect is slightly more pronounced for ‘kf_3’ and ‘kr_3’.

Furthermore, increasing ‘kf_4’ increases the flux towards mitochondria, thus reducing the lactate production rate, but is regulated by the capacity and thus has a minor effect. ‘kr_4’, on the other hand, does not show any effect, as the majority of the flux towards the mitochondria ends up accumulating in OxPP or CellComponents (Fig. S9A,B).

Variation of kf_5 and kf_6 has a very similar effect on lactate production rates, as the rate constants are equal initially. Both the rate constants reduce lactate production rates by diverting more carbon flux towards mitochondria (Fig. S10A,B).

Increasing ‘Capacity_G’ increases the glucose consumption rate and the lactate production rate, but this effect is only pronounced in the first phase. The second phase is regulated by ‘Capacity_M’, whose increase leads to reduced lactate production by promoting more carbon flux uptake into mitochondria (Fig. S11A,B).

Variations of the modulating effects of drugs are simple to interpret, as the effects are directly proportional to the rate constants and the concentration of the drug (Fig. 5, Figs S3 and S4).

The (one at a time) sensitivity analysis is relatively simple form of metabolic control analysis and confirms that the since the model is predominantly linear, control lies in the supply of the model, but is also moderated by the ‘glycolysis and mitochondrial capacities’ [73, 74]. The branching of the model at pyruvate, with a reversible route towards the TCA, links the mitochondrial activity and cytoplasmic activity, and therefore the action of the oligomycin influences the glycolysis in the cytoplasm. The lactate feedback from the extracellular environment to the cytoplasm (Reaction 4), further facilitated by the mitochondrial capacity (model control element; Reaction 5), is required to reproduce the second phase of the response. Both of these aspects introduce a degree of feedback, although not as complex as other examples of nonlinear feedback for unbranched pathways [75].

## Discussion

Although the glycolysis assay is limited to the pathway end‐point measurements, it provides a viable option to monitor the kinetics of cellular metabolism, *in vitro*. The assay is relatively easy to use, cheap and can be easily implemented, as it is based on simple spectrophotometry, enabling high‐throughput parallel analysis of biological samples in multiwell plates. The assay requires only 10 μL of sensor molecule solution per well for the kinetic analysis of a cell population, allowing multiple parallel measurement of different cell types, exposure conditions and/or modulators. It can be used in non‐TRF intensity mode on some plate readers with some signal optimisation, but provides best‐sensitivity with the time‐gated/time‐resolved fluorescence (TGF/TRF) modes, as the emission of the sensor molecule in the assay is long‐lived, compared with any shorter lived background fluorescence. The assay is advertised as having best performance using the dual‐read TRF mode, but a single‐read TRF is sufficient to obtain well‐resolved ECA curves, as illustrated in Fig. 2. The excitation (Ex) wavelength for the sensor molecule ranges from 340 to 410 nm with a peak ranging from 360 to 380 nm, and the emission (Em) has sharp peaks at 590, 615 and 690 nm, providing additional flexibility with Ex/Em filter‐based spectrophotometers, with limited choice of filters. This feature was leveraged in this study to perform the experiment at an emission wavelength of 595 nm rather than the recommended 615 nm. Additionally, it was observed that a flat‐bottomed white or transparent, rather than black plate, provided the highest signal intensity, allowing a greater statistically significant resolution of the ECA curves (data not shown). To avoid condensation, the experiment was performed without the plate cover.

It should be noted that, while lactate, as the end‐product of the glycolysis pathway, is considered to be the primary source of ECA, it has been reported that CO_2_, a by‐product of cell mitochondrial respiration, also has a significant contribution [22]. The glycolysis assay can be coupled with the oxygen consumption assay to monitor the aerobic side of the cellular metabolism to determine its contribution to the monitored ECA. However, as this study aims to explore the potential of the commercially available glycolysis assay alone the cells were starved initially for 2 h in glucose free respiration buffer, which depletes their stored energy and therefore promotes the glycolysis pathway over mitochondrial energy production as a response to the AMPK signalling mechanism (Materials and methods section, Fig. 1). The initial spike in the ECA signal due to cellular carbon reserves was also eliminated due to cell starvation (Fig. S1, Fig. 2).

The assay showed clear statistically significant differences between the responses of the cell lines to the addition of glucose, with and without modulating factors (Fig. 2). The ECA response for the A549 and LLCMK2 cell lines increased initially up to a saturation point, before subsequently decreasing. The same effect was replicated in their stimulated conditions (Fig. 2B, Fig. S2A). Cell starvation and the stimulation by oligomycin during the modulations eliminated the possibility of ECA by mitochondrial respiration so the primary extracellular acidifier remained lactate and reduction in acidification indicates its uptake by the cell, as the sensor molecule is not consumed during the experiment. This metabolic change can be used to identify two metabolic phases, the first one of high glucose consumption and lactate production while the TCA cycle remains mostly dormant, the second characterised by high lactate uptake, possibly due to glucose exhaustion, which is catabolised in the TCA and/or anabolised in gluconeogenesis.

The timescale of the measurement is an important consideration when it comes to the kinetics of the glycolysis assay. Although the respiration buffer is capable of supporting cellular metabolism in eukaryotic cells for the duration required to study the effect of individual carbon sources, it cannot support proper cell growth, as would occur in a nutrient rich culture medium for a prolonged period. Cell culture media show large absorbance in the UV region and the phenol red, usually included in the medium as a pH indicator, has Ex/Em at 400–500/500–600 nm wavelengths [76]. Neither interfere with the Ex/Em wavelengths of the sensor molecule in the glycolysis assay [77], however, and thus, the assay could be conducted in full, unbuffered nutrient rich culture medium, as described in the user manual. Nevertheless, the respiration buffer is recommended for monitoring the glycolysis kinetics as it is a mixture of several salts and can be simply modified to study the metabolic kinetics using a single carbon source. Furthermore, eukaryotic cell culture requires moisture and CO_2_ regulation as well as temperature control, which are not normally available in commonly available spectrophotometers. These factors limit the experimental duration to the recommended time of ~90 min, although this is not sufficient to capture the transition between the different phases of cellular metabolism observed here; possibly the reason why the metabolic phases generally not being described in nondose‐dependent studies. This limitation can be overcome by amending the experimental design, for example by cell starvation, as used in this study, which enhances the cellular glucose metabolic rate such that the two phases can be observed. For a shorter experimental duration, moisture control can be neglected as the evaporation rate is not high and the lack of CO_2_ regulation is rather beneficial for the glycolysis assay as the sensor molecule is sensitive to CO_2_, which can dissolve in the medium. Temperature control is a must, in both measurement scenarios, however, and lack of it provides erogenous results (data not shown).

The regulation of the experimental conditions by the addition of glucose and modulating drugs effectively tunes the cellular glycolysis rate from high, medium to low (Fig. 2B). However, the simple end‐point assay is limited in sensitivity to differentiate the intermediate levels of cellular responses, for example, the effect of varying concentrations of the carbon source or of the modulator drugs, and therefore to probe the subtleties of the kinetic metabolic pathways underpinning the ECA response. The (already simplified) glucose uptake and metabolic pathway system of Fig. 1 can be readily modelled [78, 79], and even further simplified, as shown in Fig. 3. An optimised model to simulate the experimental ECA response can further enhance the insight into the evolution of the cascading processes with easy to understand graphical representation. A mathematical model can further overcome the limitation of sensitivity to simulate the metabolic kinetics, for instance at varying modulator concentrations, lower cell density up to a single cell resolution, effects of gene knockout or over expression on the metabolic kinetics, etc. Ultimately, a perfectly optimised mathematical model should be capable of simulating any desired effect, limited by the user's imagination.

Although being an end‐point assay, the ECA response is a manifestation of the kinetic evolution of the cascading intermediate steps and the balance between the anaerobic and aerobic energy production pathways of cell metabolism. These underpinning insights into the cascading steps of a pathway were decoded using a simplistic kinetic mathematical model based on ODEs. There are several strategies for modelling a metabolic pathway, but the rate equations, such as the fluxomics approach, directly represent the phenotypic dynamics as a result of the interplay of several omics. In the phenomenological rate equation approach, each term in the ODE represents a step in the reaction pathway, and so it is easy to relate the equations to the graphical model. Each term in the rate equation is dependent on the substrate concentration and a rate constant, which defines its evolution as a function of time. Mathematical modelling can be challenging to novices, but simbiology provides ample documentation to grasp the modalities along with some modelling examples. It provides a user‐friendly graphical interface, which allows modelling based on graphics, while the mathematical equations governing the reactions are added in the background. The tool allows the user to interact with the model while it is performing the simulation, for precise model optimisation. It also has inbuilt functionalities for dose‐dependent, time‐dependent, concentration‐dependent, etc. simulations along with several options for data fitting, all of which can be conducted without any coding knowledge. The simulations are speedy and often prompt the user if some modelling criteria are not met. The model might not prompt when the model is over‐defined and takes about a minute or two to run a defined number of permutations before prompting simulation failure. As an alternative, the open‐source celldesigner [40, 59] tool allows a few more functionalities in defining the model parameters, being a tool specifically designed for the purpose, although it has limited functionality in terms of data fitting and overlaying and is prone to crashing if the model is incorrectly defined and simulated.

In this study, the motto of modelling was to keep the model as simple as possible while making it as complex as required. Several models for glucose catabolism or glycolysis, using ODE's similar to the model developed in this study, can be found, for example, in the BioModels repository, which vary in their complexities and sophistication [80, 81, 82, 83]. These models could not, however, be easily adapted to simulate the experimental ECA responses, as they were not developed specifically to simulate the end‐point kinetics and do not demonstrate similar interaction of cytoplasmic and mitochondrial counterparts necessary to simulate the two metabolic phases. Hence, the 10‐step glycolysis pathway was reduced to a few regulatory steps required for modelling the observed ECA evolution, and the aerobic part of the model was reduced even further, describing the entire TCA cycle and the OxPP as a single reaction, as the objective was limited to providing insight into the glycolysis pathway kinetics (Figs 1 and 3). The capacities incorporated in the model (Capacity_G, Capacity_M) limited the amount of carbon that can flow into the cell or the mitochondria, even if the extracellular concentrations vary with time. The extracellular glucose concentration, initially very high, gradually decreases in a regulated fashion due to the glycolysis capacity (Capacity_G) and similarly the mitochondrial capacity (Capacity_M) caps the amount of pyruvate that can enter the mitochondria at any time. This is key to the observation of the two metabolic phases in a single model without varying the rate constants with time. Optimising the model to simulate the metabolic kinetics of three different cell lines allowed quantitative comparison of key stages of their respective metabolic pathways. The numerical model developed in this study is a simplistic platform for comparing different cellular phenotypes in mathematical terms along with predicting the underlying processes, which aid further interpretations and experimentation. Thus, the model specifically incorporates only the metabolic counterparts necessary to justify the experimental ECA responses. As observed in Fig. 2A, it is clear that the glycolytic rate of LLCMK2 cell line was slightly lower than that of the A549, even if the ECA response was similar. The model clarifies that the differing responses is due to a higher lactate reversibility, which can also be interpreted as a lower lactate production rate, while the rest of the parameters are constant across the cell lines (Table 3). Furthermore, the extremely slow glycolysis metabolism leading to slow ECAR in the HepG2 cell line can be linked to its lower Glucose capacity. Furthermore, the slower lactate consumption rate or the prolonged saturated phase in HepG2 is consistent with lower mitochondrial capacity (Table 3).

Including the inhibitions by the drugs at their respective nodes in the model, as described in Materials and methods section, simulated the observed experimental ECA responses for all three cell lines. As there is only one carbon source in the system initially, inhibiting the glycolysis pathway with 2DG cuts down the flow to the entire system, including lactate, as observed in the experimental ECA responses (Fig. 2B, Fig. S2). As the model actively interacts with its anaerobic and aerobic sides, an inhibition at the aerobic side by oligomycin resulted in a stimulating effect on the anaerobic side. A stimulating effect by oligomycin was added in the anaerobic side to simulate the increased glucose consumption rate as a result of activation of the AMPK signalling mechanism (Reaction 2; Table 1). In the stimulated condition, A549 and LLCMK2 ECA responses were similar, but enhanced compared with their corresponding control samples. The HepG2 cell line showed little or no effect of stimulation by oligomycin (Fig. S2B), behaviour characteristic of highly glycolytic cells or cells without mitochondria [62, 84], which already rely primarily of glycolysis for their energy. This effect was reproduced by reducing the value of inhibition/stimulation rate constant by oligomycin, which essentially represented lower drug efficacy for the cell line. Furthermore, the model can simulate the ECA responses at varying modulator concentrations, which is challenging experimentally due to low assay resolution. The assay can be used to visualise the effects of modulators/drugs on the glycolysis pathway kinetics, whereas the metabolic model can be used to quantitatively compare the drug efficacies for different cell lines, elucidating viable drug doses and their effect on the metabolism for a range of disorders linked to glycolytic kinetics, as described by Patil *et al*. [3]. Furthermore, such models may, in future, act as templates to guide the data mining of relevant characteristic signatures in label‐free analysis techniques, such as vibrational microspectroscopies, and their adaptation to monitoring metabolomic pathway, in a holistic manner [3, 85].

The study therefore demonstrates the potential of the simple commercially available glycolysis assay, which can effectively provide limited yet kinetic insight into the cellular glycolysis pathway. Furthermore, a relatively simple numerical model can greatly enhance the analytical scope of the commercially available *in vitro* assay by interpreting the underpinning reaction steps in the metabolic pathway from the end‐point kinetics and furthermore predicting the systemic behaviour under modulations, which might not be resolvable experimentally. It is acknowledged, however, that the assay itself has major limitations, in that it fails to differentiate the ECA response from several sources such as lactate or CO_2_. Furthermore, the simplified numerical model provides limited insight into the cascading processes enriched with several layers of metabolic regulations. Nevertheless the assay proves to be an easy one‐step cost‐effective go‐to approach for monitoring the glycolysis pathway kinetics under several modulations while the model aids in simulating the blindspots or the experimental limitations and comparison among diverse phenotypes numerically. The assay is suitable for large‐scale parallel studies such as drug screening, and a numerical model can augment the limited insight with some knowledge of the cellular mechanisms.

## Conflict of interest

The authors declare no conflict of interest.

### Peer review

The peer review history for this article is available at https://www.webofscience.com/api/gateway/wos/peer‐review/10.1002/2211‐5463.13765.

## Author contributions

NP was involved in conceptualization and writing—original draft. ZM was involved in conceptualization. HJB was involved in conceptualization, writing—original draft, supervision and funding acquisition.

## Supporting information


**Fig. S1.** Assay response measurements as a function of time (up to 180 min) representative of extracellular acidification rate (ECAR) for stimulation by oligomycin (green), and inhibition by 2‐deoxyglucose (red) for HepG2 cell line without two‐hour cell starvation. The dotted line separates the initial spike from the gradual increase in the extracellular acidification (ECA) signal. The initial spike was relatively normalised by starving the cells of carbon source (glucose) for two hours (main text Fig. 2), which led to the conclusion that the initial spiking in the ECA was due to the cellular carbon reserves.
**Fig. S2.** Assay response measurements as a function of time (up to 140 min) representative of extracellular acidification rate (ECAR) for LLCMK2 (A) and HepG2 (B) cell lines for control (purple), stimulated by oligomycin (green) and inhibition by 2‐deoxyglucose (red).
**Fig. S3.** Pathway modulation using (A) oligomycin and (B) 2DG for the LLCMK2 cell line optimised model: The experimental extracellular acidification (ECA) results are overlaid on top of the simulated lactate (orange) curves under different modulation conditions. The evolution of extracellular glucose (dark blue) and the model end products ETC (green) and CellComponents (light blue) are also shown. The extracellular glucose and model end products are shown separately for inhibition by 2deoxyglucose (2DG) in ‘C’ for better visualisation of extracellular lactate curves.
**Fig. S4.** Pathway modulation using (A) oligomycin and (B) 2DG for the HepG2 cell line optimised model: The experimental extracellular acidification (ECA) results are overlaid on top of the simulated lactate (orange) curves under different modulation conditions. The evolution of extracellular glucose (dark blue) and the model end products ETC (green) and CellComponents (light blue) are also shown. The extracellular glucose and model end products are shown separately for inhibition by 2deoxyglucose (2DG) in ‘C’ for better visualisation of extracellular lactate curves.
**Fig. S5.** Model sensitivity analysis of Glu_ex (extracellular glucose) (A); k_in (glucose uptake rate constant) (B) with 10% increment or decrement from the A549 optimised model parameter for 11 consecutive simulations (five increase and five decrease). The extracellular glucose consumption (blue) and extracellular lactate (orange) kinetics for 140 min are shown.
**Fig. S6.** Model sensitivity analysis of kf_1 (glucose to pyruvate forward rate constant) (A), kr_1 (glucose to pyruvate reverse rate constant) (B) with 10% in increment or decrement from the A549 optimised model parameter for 11 consecutive simulations (five increase and five decrease). The extracellular glucose consumption (blue) and extracellular lactate (orange) kinetics for 140 min are shown.
**Fig. S7.** Model sensitivity analysis of kf_2 (pyruvate to lactate forward rate constant) (A), kr_2 (pyruvate to lactate reverse rate constant) (B) with 10% in increment or decrement from the A549 optimised model parameter for 11 consecutive simulations (five increase and five decrease). The extracellular glucose consumption (blue) and extracellular lactate (orange) kinetics for 140 min are shown.
**Fig. S8.** Model sensitivity analysis of kf_3 (lactate to extracellular lactate forward rate constant) (A), kr_3 (lactate to extracellular lactate reverse rate constant) (B) with 10% increment or decrement from the A549 optimised model parameter for 11 consecutive simulations (five increase and five decrease). The extracellular glucose consumption (blue) and extracellular lactate (orange) kinetics for 140 min are shown in each case. Fig. S8 A and B show a high degree of similarity, as increasing or decreasing the value of the forward rate kf_3 slightly increases or decreases the lactate production rate, respectively, whereas an equivalent inverse effect is observed for variation of the reverse rate kr_3. This similarity indicates a lack of control in the lactate transport in and out of the cell (Reaction 4) since the modulation of the rate constants does not produce a significant effect, compared to similar modulation of other rate constants in the model.
**Fig. S9.** Model sensitivity analysis of kf_4 (pyruvate to TCA forward rate constant) (A), kr_4 (pyruvate to TCA reverse rate constant) (B) with 10% in increment or decrement from the A549 optimised model parameter for 11 consecutive simulations (five increase and five decrease). The extracellular glucose consumption (blue) and extracellular lactate (orange) kinetics for 140 min are shown.
**Fig. S10.** Model sensitivity analysis of kf_5 (TCA to ETC forward rate constant) (A), kf_6 (TCA to CellComponents forward rate constant) (B) with 10% increment or decrement from the A549 optimised model parameter for 11 consecutive simulations (five increase and five decrease). The extracellular glucose consumption (blue) and extracellular lactate (orange) kinetics for 140 min are shown. Variation of kf_5 and kf_6 has an identical effect on the extracellular lactate kinetics, as the rate constants are initially equal. Increasing either of the rate constants reduces lactate production rates by diverting more carbon flux towards the mitochondria, and vice versa.
**Fig. S11.** Model sensitivity analysis of Capacity_G (glycolysis pathway capacity) (A), Capacity_M (mitochondrial capacity) (B) with 10% in increment or decrement from the A549 optimised model parameter for 11 consecutive simulations (five increase and five decrease). The extracellular glucose consumption (blue) and extracellular lactate (orange) kinetics for 140 min are shown.

## Data Availability

The numerical model (MODEL2312220001) and the experimental extracellular acidification (ECA) data for A549, LLCMK2 and HepG2 cell lines, with and without modulations, are available in the BioModels repository of the European Bioinformatics Institute at https://identifiers.org/biomodels.db/MODEL2312220001.
