# Supplementary material for: Kinetic modelling of the cellular metabolic responses underpinning in vitro glycolysis assays
Source: FEBS Open Bio. 2024 Jan 12;14(3):466–86. doi: 10.1002/2211-5463.13765 (PMC10909989; doi:10.1002/2211-5463.13765)
Supplement: Supplementary file 1 — Fig. S1. Assay response measurements as a function of time (up to 180 min) representative of extracellular acidification rate (ECAR) for stimulation by oligomycin (green), and inhibition by 2‐deoxyglucose (red) for HepG2 cell line without two‐hour cell starvation. The dotted line separates the initial spike from the gradual increase in the extracellular acidification (ECA) signal. The initial spike was relatively normalised by starving the cells of carbon source (glucose) for two hours (main text Fig. 2), which led to the conclusion that the initial spiking in the ECA was due to the cellular carbon reserves. Fig. S2. Assay response measurements as a function of time (up to 140 min) representative of extracellular acidification rate (ECAR) for LLCMK2 (A) and HepG2 (B) cell lines for control (purple), stimulated by oligomycin (green) and inhibition by 2‐deoxyglucose (red). Fig. S3. Pathway modulation using (A) oligomycin and (B) 2DG for the LLCMK2 cell line optimised model: The experimental extracellular acidification (ECA) results are overlaid on top of the simulated lactate (orange) curves under different modulation conditions. The evolution of extracellular glucose (dark blue) and the model end products ETC (green) and CellComponents (light blue) are also shown. The extracellular glucose and model end products are shown separately for inhibition by 2deoxyglucose (2DG) in ‘C’ for better visualisation of extracellular lactate curves. Fig. S4. Pathway modulation using (A) oligomycin and (B) 2DG for the HepG2 cell line optimised model: The experimental extracellular acidification (ECA) results are overlaid on top of the simulated lactate (orange) curves under different modulation conditions. The evolution of extracellular glucose (dark blue) and the model end products ETC (green) and CellComponents (light blue) are also shown. The extracellular glucose and model end products are shown separately for inhibition by 2deoxyglucose (2DG) in ‘C’ for better visualisation of e [file FEB4-14-466-s001.docx]

**Kinetic modelling of the cellular metabolic responses underpinning in vitro glycolysis assays**

**Nitin Patil^1,2^*, Zohreh Mirveis^1,2^, Hugh J. Byrne^1^**

^1^FOCAS Research Institute, TU Dublin, City Campus, Camden Row, Dublin 8, Ireland

^2^School of Physics, Optometric and Clinical Sciences, TU Dublin, City Campus, Grangegorman, Dublin 7, Ireland

*Corresponding Author: Nitin Patil (D21127295@mytudublin.ie)

**Supplementary Material**


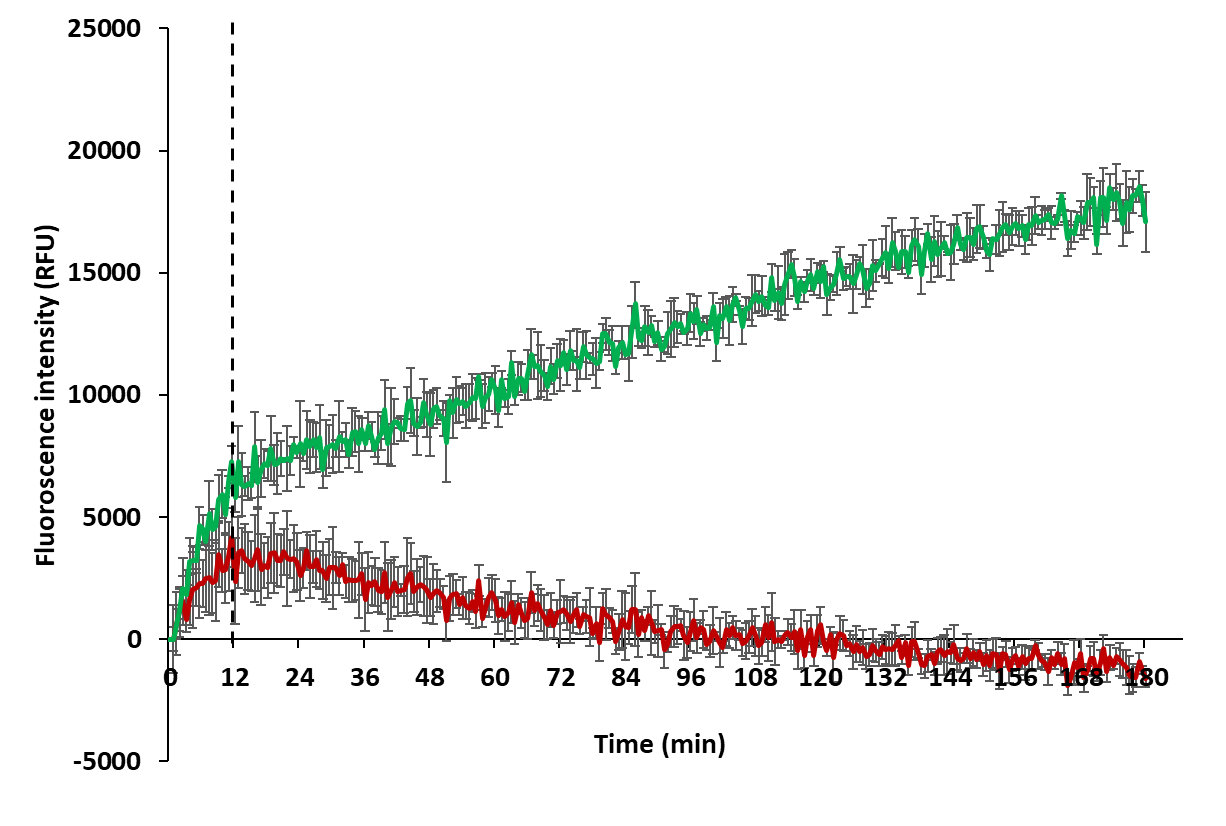


Figure S1: Assay response measurements as a function of time (up to 180 minutes) representative of extracellular acidification rate (ECAR) for stimulation by oligomycin (green), and inhibition by 2-deoxyglucose (red) for HepG2 cell line without two-hour cell starvation. The dotted line separates the initial spike from the gradual increase in the extracellular acidification (ECA) signal. The initial spike was relatively normalised by starving the cells of carbon source (glucose) for two hours (main text figure 2) which led to the conclusion that the initial spiking in the ECA was due to the cellular carbon reserves.

**
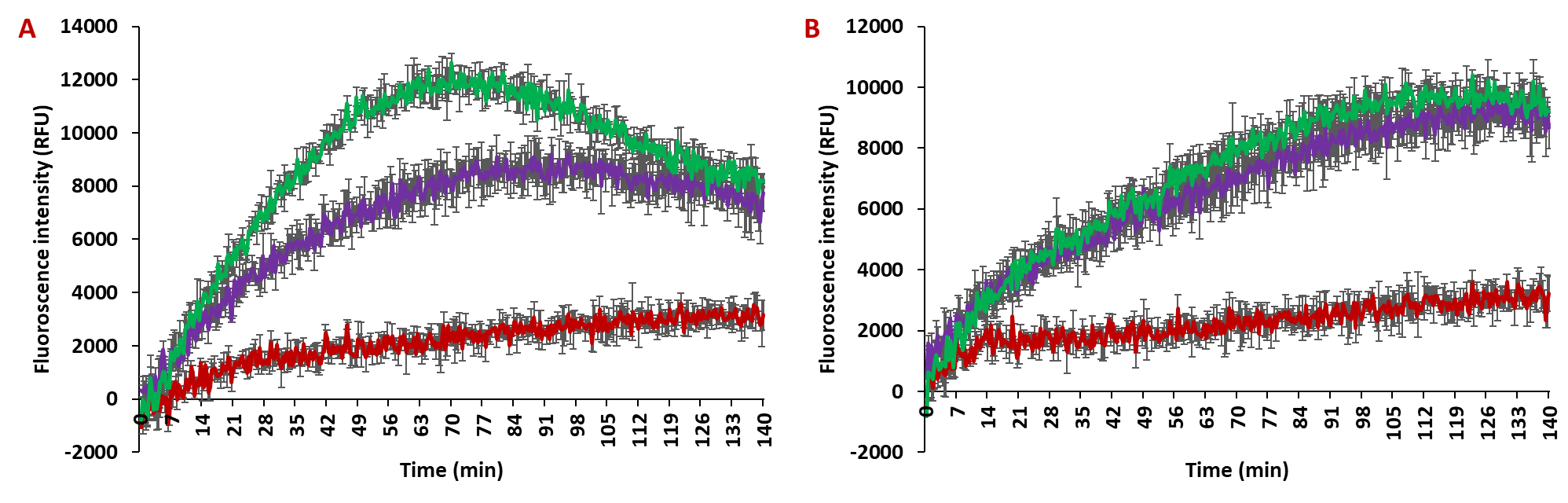
**

Figure S2: Assay response measurements as a function of time (up to 140 minutes) representative of extracellular acidification rate (ECAR) for LLCMK2 (A) and HepG2 (B) cell lines for control (purple), stimulated by oligomycin (green), and inhibition by 2-deoxyglucose (red).


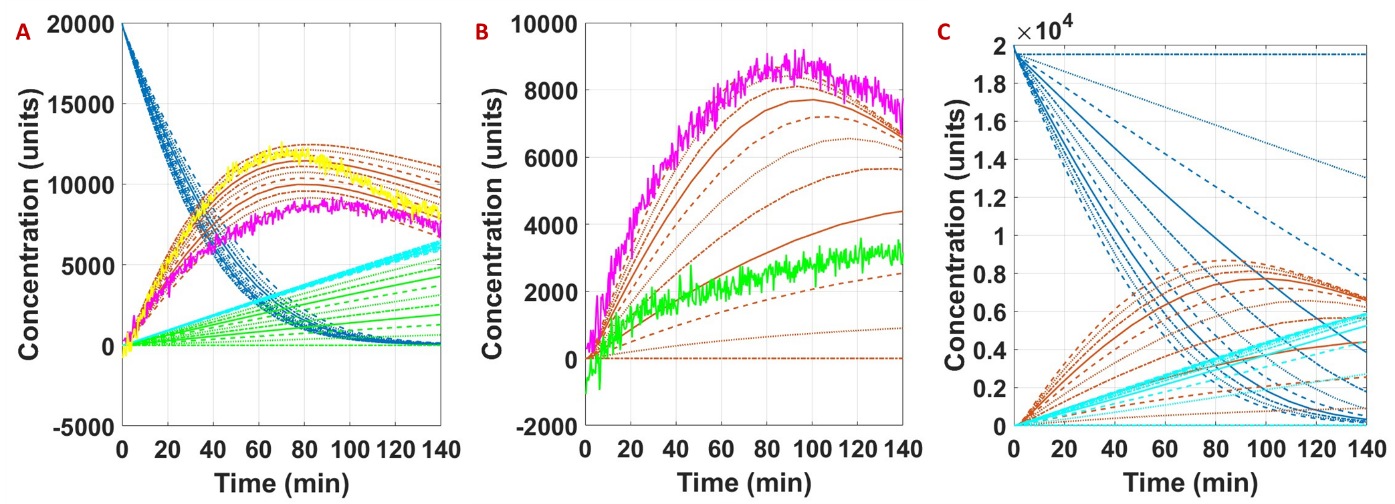


Figure S3: Pathway modulation using (A) oligomycin and (B) 2DG for the LLCMK2 cell line optimised model: The experimental extracellular acidification (ECA) results are overlaid on top of the simulated lactate (orange) curves under different modulation conditions. The evolution of extracellular glucose (dark blue) and the model end products ETC (green) and CellComponents (light blue) are also shown. The extracellular glucose and model end products are shown separately for inhibition by 2deoxyglucose (2DG) in ‘C’ for better visualisation of extracellular lactate curves.


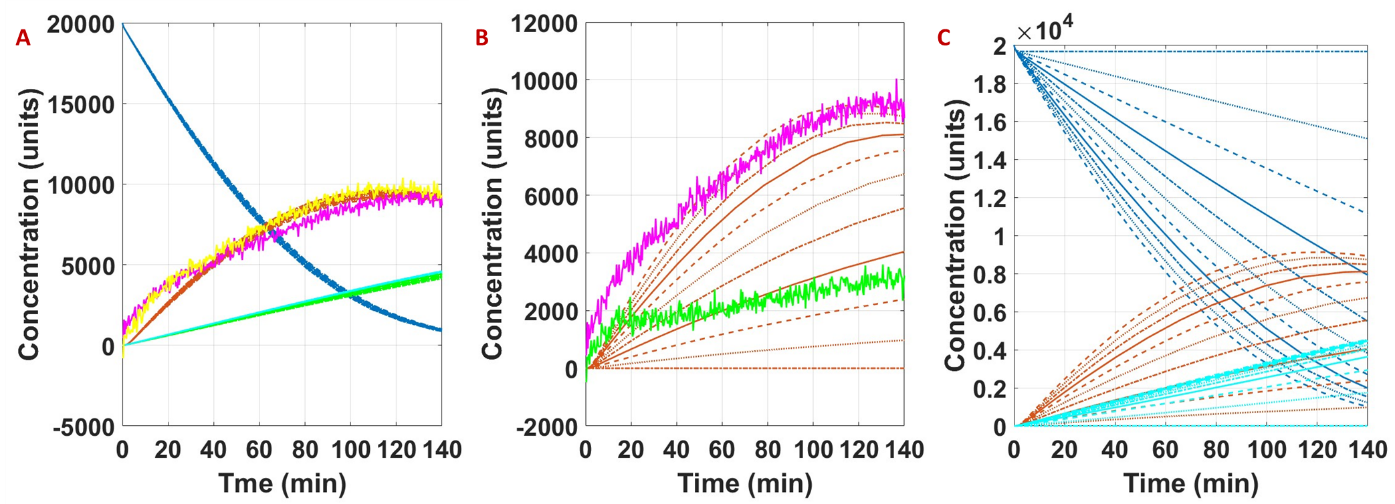


Figure S4: Pathway modulation using (A) oligomycin and (B) 2DG for the HepG2 cell line optimised model: The experimental extracellular acidification (ECA) results are overlaid on top of the simulated lactate (orange) curves under different modulation conditions. The evolution of extracellular glucose (dark blue) and the model end products ETC (green) and CellComponents (light blue) are also shown. The extracellular glucose and model end products are shown separately for inhibition by 2deoxyglucose (2DG) in ‘C’ for better visualisation of extracellular lactate curves.


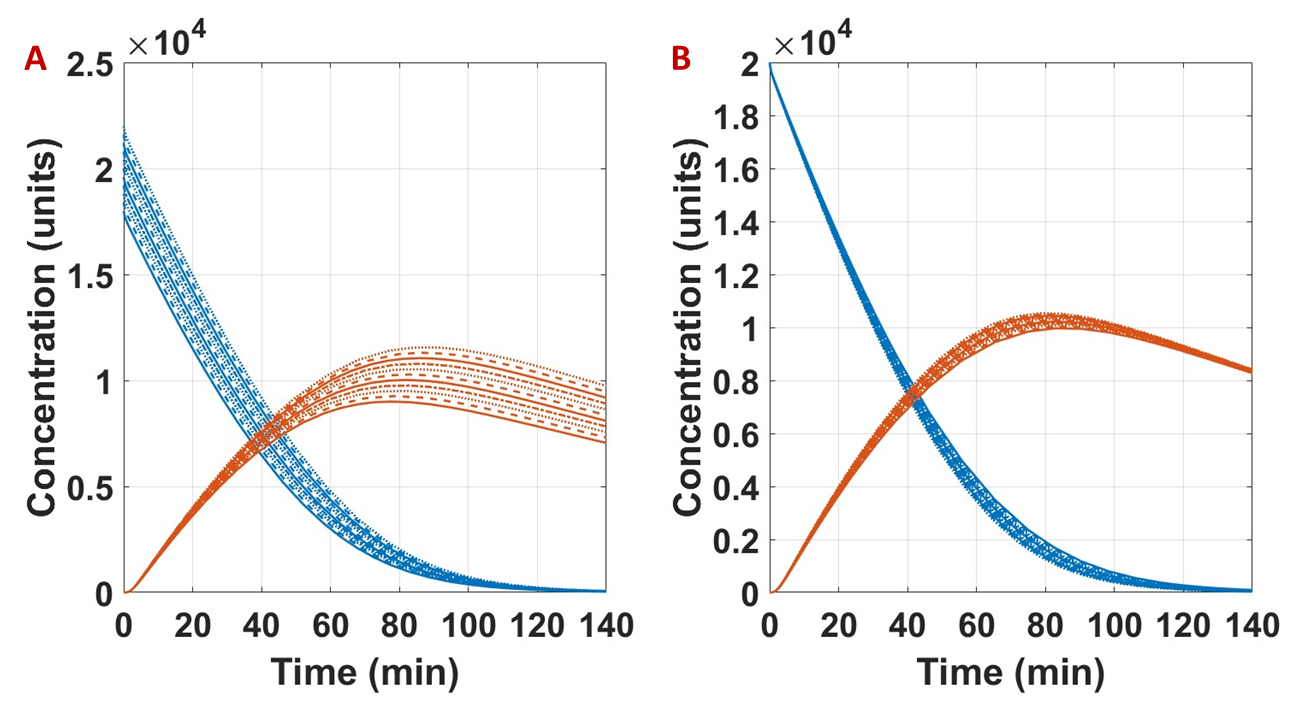


Figure S5: Model sensitivity analysis of Glu_ex (extracellular glucose) (A); k_in (glucose uptake rate constant) (B) with 10% increment or decrement from the A549 optimised model parameter for eleven consecutive simulations (five increase and five decrease). The extracellular glucose consumption (blue) and extracellular lactate (orange) kinetics for 140 minutes are shown.


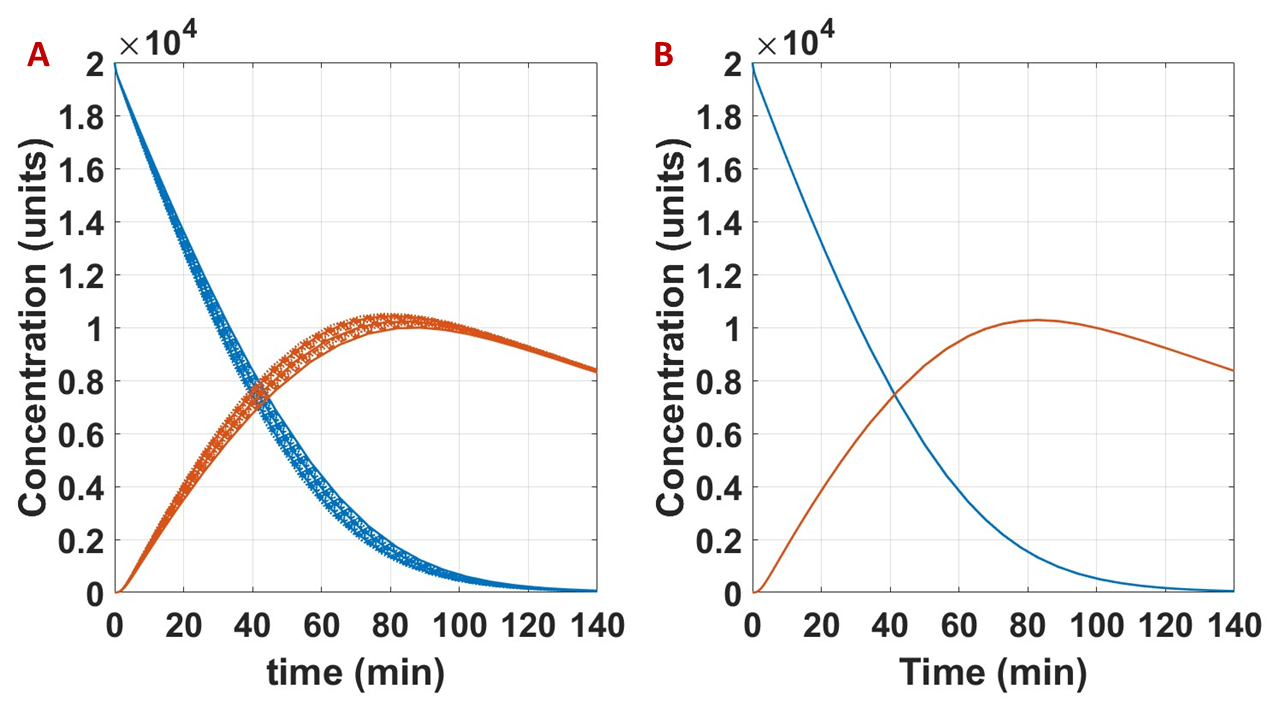


Figure S6: Model sensitivity analysis of kf_1 (glucose to pyruvate forward rate constant) (A), kr_1(glucose to pyruvate reverse rate constant) (B) with 10% in increment or decrement from the A549 optimised model parameter for eleven consecutive simulations (five increase and five decrease). The extracellular glucose consumption (blue) and extracellular lactate (orange) kinetics for 140 minutes are shown.


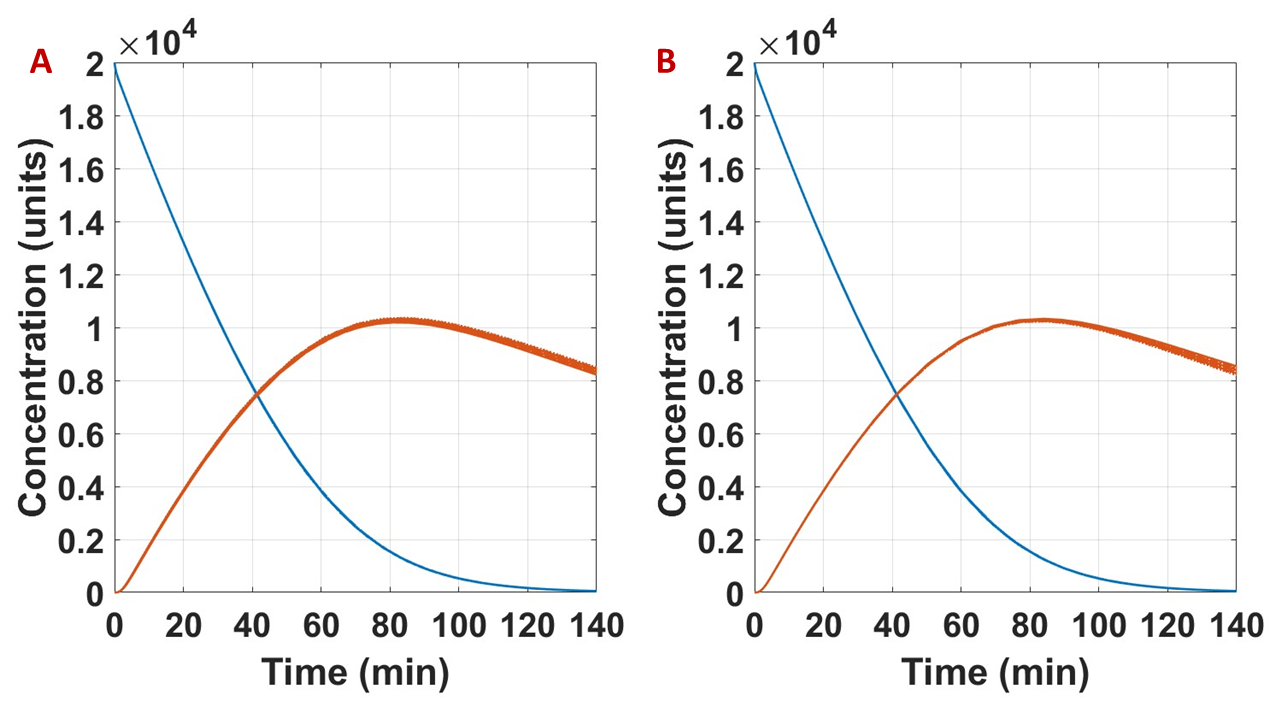


Figure S7: Model sensitivity analysis of kf_2 (pyruvate to lactate forward rate constant) (A), kr_2 (pyruvate to lactate reverse rate constant) (B) with 10% in increment or decrement from the A549 optimised model parameter for eleven consecutive simulations (five increase and five decrease). The extracellular glucose consumption (blue) and extracellular lactate (orange) kinetics for 140 minutes are shown.


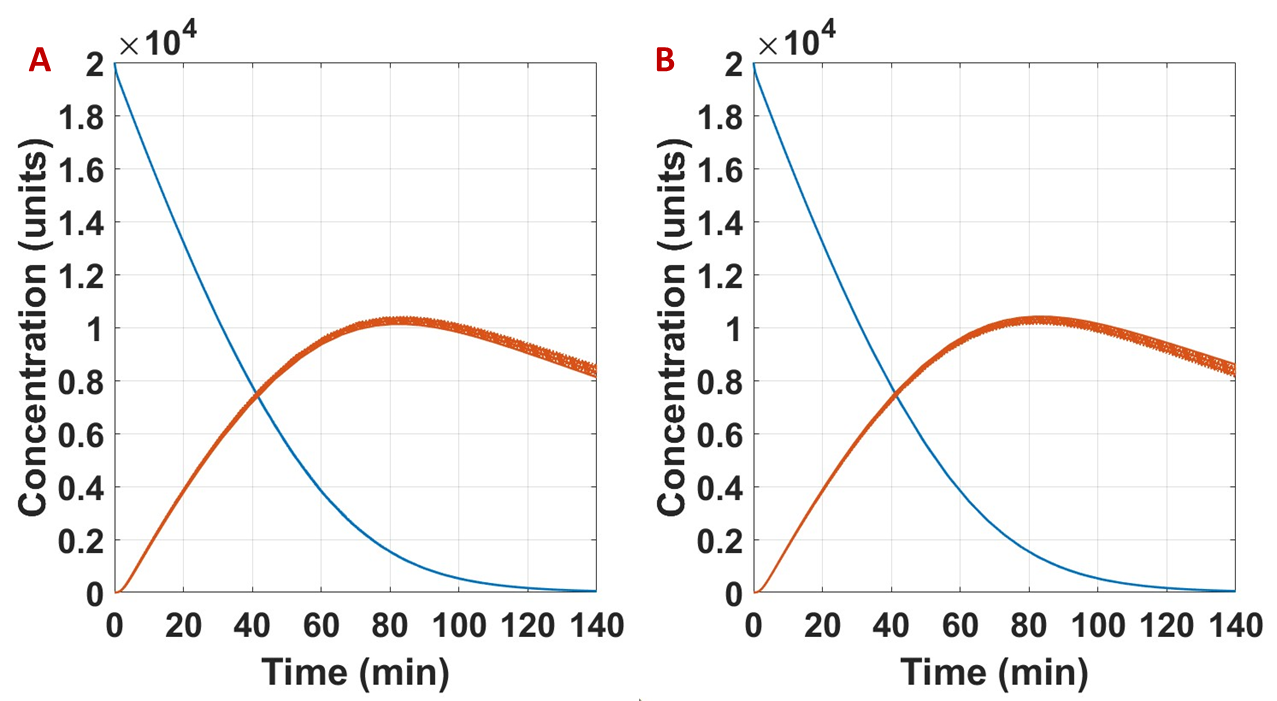


Figure S8: Model sensitivity analysis of kf_3 (lactate to extracellular lactate forward rate constant) (A), kr_3 (lactate to extracellular lactate reverse rate constant) (B) with 10% increment or decrement from the A549 optimised model parameter for eleven consecutive simulations (five increase and five decrease). The extracellular glucose consumption (blue) and extracellular lactate (orange) kinetics for 140 minutes are shown in each case. Figures S8 A and B show a high degree of similarity, as increasing or decreasing the value of the forward rate kf_3 slightly increases or decreases the lactate production rate, respectively, whereas an equivalent inverse effect is observed for variation of the reverse rate kr_3. This similarity indicates a lack of control in the lactate transport in and out of the cell (reaction 4) since the modulation of the rate constants does not produce a significant effect, compared to similar modulation of other rate constants in the model.


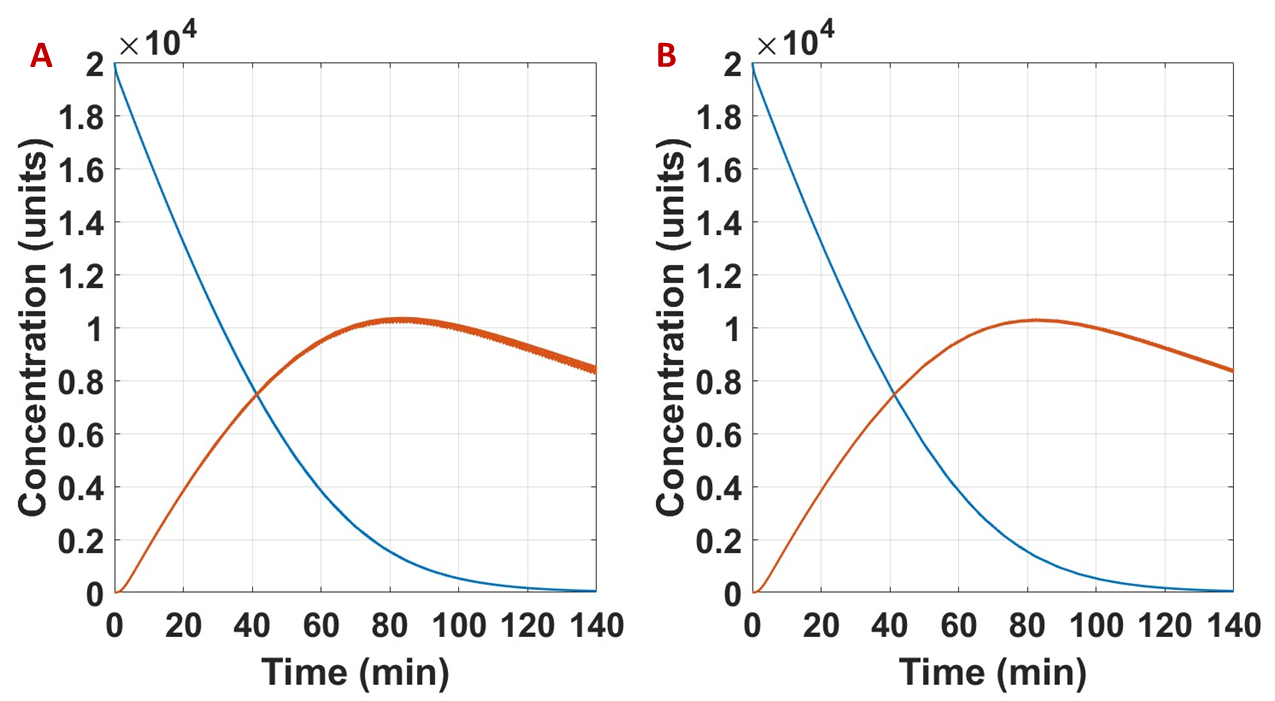


Figure S9: Model sensitivity analysis of kf_4 (pyruvate to TCA forward rate constant) (A), kr_4 (pyruvate to TCA reverse rate constant) (B) with 10% in increment or decrement from the A549 optimised model parameter for eleven consecutive simulations (five increase and five decrease). The extracellular glucose consumption (blue) and extracellular lactate (orange) kinetics for 140 minutes are shown.


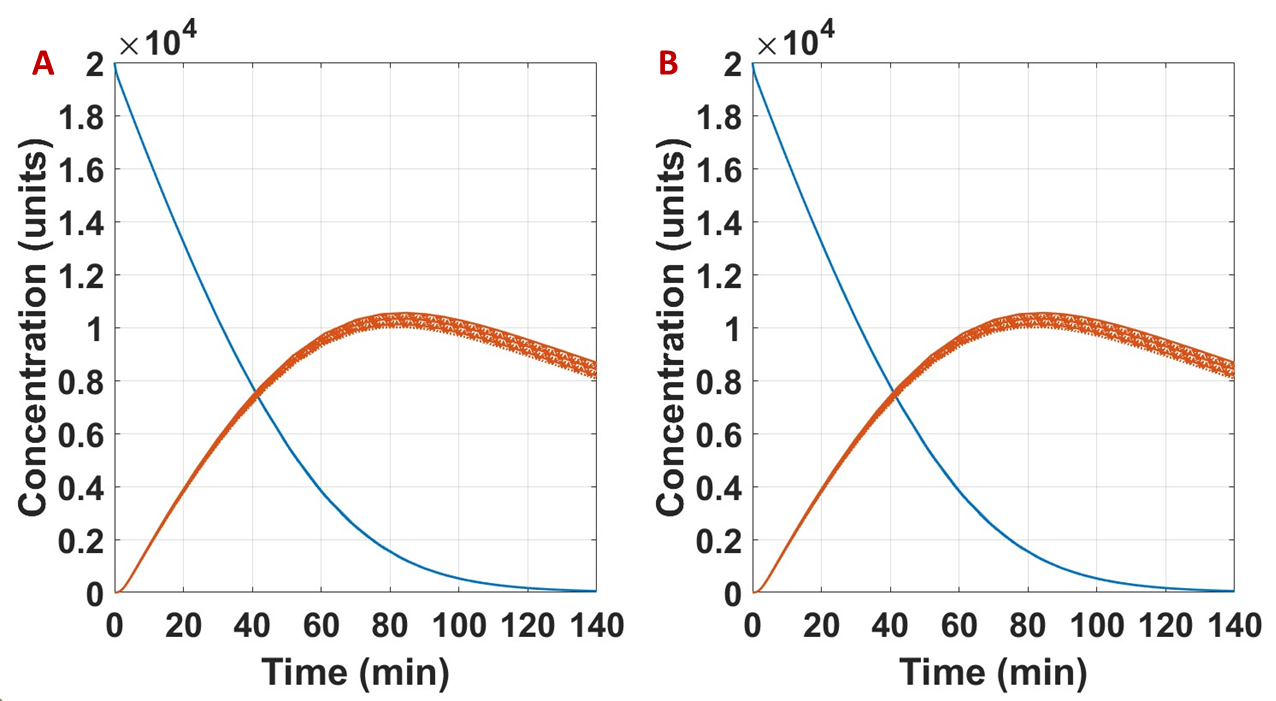


Figure S10: Model sensitivity analysis of kf_5 (TCA to ETC forward rate constant) (A), kf_6 (TCA to CellComponents forward rate constant) (B) with 10% increment or decrement from the A549 optimised model parameter for eleven consecutive simulations (five increase and five decrease). The extracellular glucose consumption (blue) and extracellular lactate (orange) kinetics for 140 minutes are shown. Variation of kf_5 and kf_6 has an identical effect on the extracellular lactate kinetics, as the rate constants are initially equal. Increasing either of the rate constants reduces lactate production rates by diverting more carbon flux towards the mitochondria, and vice versa.


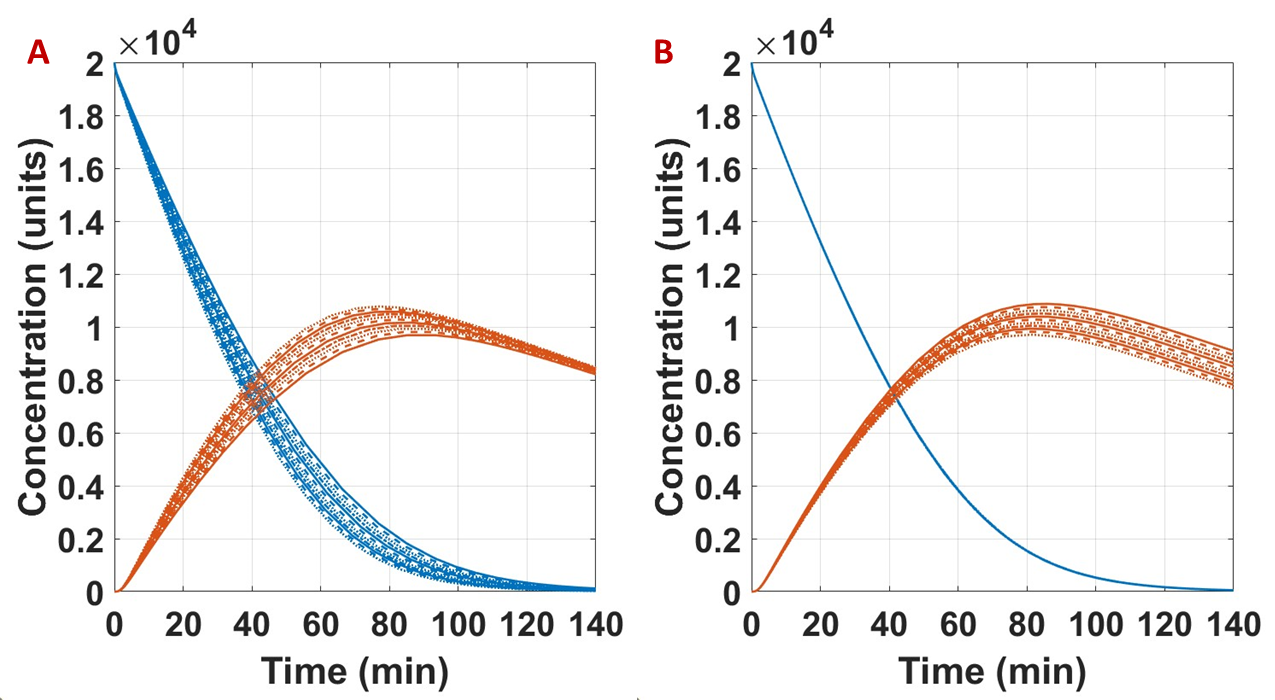


Figure S11: Model sensitivity analysis of Capacity_G (glycolysis pathway capacity) (A), Capacity_M (mitochondrial capacity) (B) with 10% in increment or decrement from the A549 optimised model parameter for eleven consecutive simulations (five increase and five decrease). The extracellular glucose consumption (blue) and extracellular lactate (orange) kinetics for 140 minutes are shown.
